# Evolution and distribution of rabies viruses from a panorama view

**DOI:** 10.1128/spectrum.05257-22

**Published:** 2023-09-05

**Authors:** Gen Li, Yue Zhang, Hong-Ling He, Chang-Yi Chen, Xin Li, Yu Xiao, Zhi-Bin Yan, Ying Chu, Jun Luo, Xiao-Feng Guo

**Affiliations:** 1 College of Veterinary Medicine, South China Agricultural University, Guangzhou, China; University of Prince Edward Island, Charlottestown, Prince Edward Island, Canada

**Keywords:** rabies virus, evolution, distribution, transmission, mutation

## Abstract

Rabies kills more than 59,000 people annually, mainly in developing countries. Previous studies on the evolution and distribution of rabies viruses (RABVs) were scattered. Here, we explore the evolution and distribution of this deadly virus from a novel panorama view. Multiple bioinformatic software tools were employed to analyze the phylogenetic diversity, evolution, spatiotemporal, and distribution of RABVs. The analyses were based on 1,202 qualified full-length genomes of RABVs and numerous literatures. Of the 10 distinct phylogenetic clades of RABV that we identified, more frequent intra- and inter-clade recombination occurs in the sequences of Asian-SEA, Arctic, and Cosmopolitan clades isolated from China, while according to existing sequence information, RABV might originate from bats (posterior probability, PP = 0.75, PP = 0.60 inferred from N and L genes, separately) in North America (PP = 0.57, PP = 0.62 inferred from N and L genes, separately). Due to the difference in evolutionary rate of N (2.22 × 10^−4^ subs/site/year, 95% HPD 1.99–2.47 × 10^−4^ subs/site/year) and L genes (1.67 × 10^−4^ subs/site/year, 95% HPD 1.59–1.74 × 10^−4^ subs/site/year), the root age was 1,406.6 (95% HPD 1,291.2–1,518.2) and 1,122.7 (95% HPD 1,052.4–1,193.9) inferred from N and L genes, separately. Among other findings, Mephitidae plays an important role in the interspecific transmission and communication of RABV, which we found tends to spread to populations genetically proximate to the host. We also identified amino acids under positive selection in different genes of different clades as well as single nucleotide variation sites important for different lineages.

**IMPORTANCE:**

Rabies virus is widely distributed all over the world, and wild animals are its largest potential reservoir. Our study offers a panorama view about evolution and distribution of rabies viruses and emphasizes the need to monitor the transmission dynamics of animal rabies.

## INTRODUCTION

Rabies is a zoonotic disease caused by the rabies virus (RABV), which can infect nearly all warm-blooded animals, including humans, with a fatality rate of almost 100%. More than 59,000 people die from rabies each year, and more than 95% of them are from developing countries in Africa and Asia (1 – 3). Therefore, rabies is an infectious disease of poverty (4).

Dogs are the dominant host of RABV worldwide, and approximately 99% of human rabies cases are due to bites from infected dogs (5). International organizations, including the World Health Organization (WHO), the UN Food and Agriculture Organization (FAO), the World Organization for Animal Health (WOAH), and the Global Alliance for Rabies Control (GARC), are supporting countries to eliminate dog-mediated rabies by 2030 (https://www.who.int/news-room/commentaries/detail/new-global-strategic-plan-to-eliminate-dog-mediated-rabies-by-2030).

RABV is a lipid-enveloped virus with bullet-shaped morphology and a negative-sense, single-stranded, and non-segmented RNA genome. Its genome (approximately 12 kb) consists of five structural genes encoding the viral nucleoprotein (N), phosphoprotein (P), matrix protein (M), glycoprotein (G), and polymerase (L) from the 3′ terminal to the 5′ terminal (6).

RABV belongs to the genus Lyssavirus in the family *Rhabdoviridae*. Currently, Lyssavirus contains 17 species (https://ictv.global/report/chapter/rhabdoviridae/rhabdoviridae) and circulate among bats (order *Chiroptera*) and carnivores (order *Carnivora*), although they can infect all warm-blooded animals (7). Lyssavirus species have been classified in phylogenetics into RABV and RABV-related species or viruses (7), and lyssaviruses have been classified into Chiropteran lyssaviruses (including RABV-related viruses and some clades of RABV mainly circulating in bats) and Carnivoran lyssaviruses (i.e., some clades of RABV mainly circulating in carnivores). The time of carnivoran rabies switching from chiropteran lyssaviruses was between 542 and 1,113 by studying 36 carnivoran and 17 chiropteran lyssaviruses representing the main genotypes and variants of the viruses (7, 8).

RABV is the type species of the genus Lyssavirus and can be divided into eight clades, including Africa-2, Africa-3, Arctic-related, Asian, Bats, Cosmopolitan, Indian sub, and RAC-SK (8). Of these clades, Africa-2, Africa-3, Arctic-related, Asian, Cosmopolitan, and Indian-sub belong to Carnivoran lyssaviruses, which are also termed as dog-related clades. The clades of Bats and RAC-SK belong to Chiropteran lyssaviruses, which are also termed as bat-related clades. Troupin et al. utilized five concatenated genes of RABV to analyze the time of origin, with the tree root between 1,308 and 1,510, the Afrcia-2 between 1,740 and 1,852, the Africa-3 between 1,710 and 1,815, the Asia between 1,525 and 1,677, the Arctic-related between 1,725 and 1,815, Cosmopolitan between 1,687 and 1,773, and Indian-sub between 1,733 and 1,840 (8 – 10). However, the phylogeographic analyses and inferences were made only for dog-related RABV, excluding the two bat-related clades, Bats and RAC-SK. Recently, Kathryn et al. proposed a lineage classification software for RABV, called MADDOG, combined with a new rabies virus sequence data resource (RABV-GLUE) to track lineage dynamics and to make the classification more refined and convenient (9). Previous studies and tools have advanced the process of studying RABV and have given researchers much inspiration. However, the sequence information analyzed by pioneers focused on one or two hosts or one city or one country, and increasing information on RABV full-length genomes become available with the improvement of sequencing technology (11 – 15).

Therefore, a comprehensive analysis of the spatial and temporal spread of RABV is not currently available. To fill the gap, we adopted the latest RABV full-length genome data set, covering 62 countries and 69 host species, including related-dog and related-bat, to provide a panorama view about evolution and distribution of RABV.

## MATERIALS AND METHODS

### Sequence data set

Full-length genome sequences of RABVs were retrieved from GenBank available before 1 October 2022. All the sequences without the information on collection countries or collection years were removed. The remaining sequences, one of which was reported by our laboratory (GenBank accession number JX088694), were exported to PhyloSuite (version 1.2.2) to remove identical sequences (16). The remaining 1,202 sequences were referred to as qualified sequences in this study and were aligned using the MAFFT software (version 7.149) (17) (Table S1). Amino acid residues were numbered as per the RABV genomic sequence with the accession number NC001542.

### Phylogenetic analysis of genomic sequences

The best-fitting nucleotide substitution model [i.e., the one of the lowest Bayesian information criterion (BIC) value] was determined to be the general time-reversible substitution model with empirical base frequencies and relaxing gamma-distributed rate heterogeneity (GTR + F + R9), using the ModelFinder software (version 1.6.8) (18). The phylogenetic signal was assessed using likelihood-mapping, with the best-fitting model and 30,050 randomly selected quartets (19). Phylogenetic relationships were calculated using the IQ-Tree software (version 1.6.8) as per the best-fitting nucleotide substitution model and the maximum-likelihood (ML) method. Bootstrap values were calculated using 1,000 replicates (20). The final results were visualized in the Interactive Tree of Life (21).

### Principal component analysis of genomic sequences

We used SNP-sites (version 2.5.1) to identify single nucleotide variations (SNVs) and Plink (version 1.90) to filter those SNVs occurring in less than 5% of sequences (22, 23). We performed a principal component analysis to visualize the SNV analysis results using the smartpca package and the packages ggplot2 and RColorBrewer in the R, to further validate the analysis results (24, 25).

### Recombination analysis of genomic sequences

Full-length genome sequences of RABV were analyzed to detect potential recombination events using the software tools RDP4 (version 4.101) and GARD (http://www.datamonkey.org/gard) (26). Recombination detected by at least four of the seven methods, including RDP, GENECONV, Bootscan, MaxChi, Chimaera, SisScan, and 3Seq, was considered as true recombination with a *P*-value threshold of 0.05 (27, 28).

### Temporal signal analysis of genomic sequences

The software tools TempEst (version 1.5.3) based on a strict molecular clock and TreeTime (version 0.9.1) based on both a strict and relaxed molecular clock were used to assess the temporal signals of the evolution of RABV genomic sequences (29, 30). We used those three modes to detect the correlations, the evolutionary rates, and the most recent common ancestors. To beget sampling bias in subsequent analyses, only the remaining five clades were analyzed in this study. Moreover, we removed duplicate sequences, recombinant sequences, and deviating time sequences to fit the molecular clock model.

The best-fitting nucleotide substitution models were GTR + F + G4 for the N gene and TVM + F + G4 for the L gene. In a subsequent step, we combined the tree prior and the molecular clock model to obtain the value of marginal likelihood estimation by analyzing path sampling and stepping-stone sampling (https://beast.community/bets_tutorial).

### Evolutionary analysis and phylogeographic inference

The Bayesian Markov Chain Monte Carlo (MCMC) method in the BEAST (version 1.10.4) package, along with BEAGLE, was used with the suitable model (31, 32). For five independent data sets with 100 million steps, multiple runs of MCMC were combined using LogCombiner (version 1.10.4) and 500 million steps for each set, with sampling every 10,000 steps and a final sample size exceeding 10,000. Tracer software (version 1.7) was used to confirm that all parameter estimates yielded effective sampling sizes greater than 200 and that 10% of the total chain length had been burned up to reduce the influence of the initial value (33). The final Bayesian maximum clade credibility (MCC) tree was generated by TreeAnnotator (version 2.6.3) and visualized in Figtree (version 1.4.4) (34).

The Bayesian stochastic search variable selection (BSSVS) traits model was used to analyze phylogeographic inference, after which the SpreaD3 (version 0.9.6) application within BEAST was used to develop interactive visualizations of the dispersal process across time (35).

### Gene mutation and protein function analysis

Tassel (version 5.2.82) was used for a genome-wide association study (GWAS), while LDBlockShow (version 1.35) was applied to analyze and visualize (36, 37). Positive selection was detected by Datamonkey (http://www.datamonkey.org). Four methods were employed, namely the mixed-effects model of evolution (MEME), fixed-effects likelihood testing (FEL), single-likelihood ancestor counting (SLAC), and fast unconstrained Bayesian approximation (FUBAR). Codons highlighted by at least three methods were considered to be under selection. The 3D structure of the G protein was assembled and simulated by the I-TASSER service (38). The mutant amino acids were analyzed with DynaMut (https://biosig.lab.uq.edu.au/dynamut/) and visualized with VMD (http://www.ks.uiuc.edu/Research/vmd/).

## RESULTS

### Detection and distribution of RABV

The viral strain GD-SH (accession no. JX088694) was isolated from pigs suspected of having rabies and was subsequently used to infect mice. The direct immunofluorescence assay was used to identify the presence of the GD-SH strain in the infected mice (Fig. S1A). The structure of RABV is shown in Fig. S1B and C.

According to data from the NCBI, a total of 62 countries have reported that 69 host species can be infected with RABV across five continents, excluding Oceania and Antarctica (Fig. S2A). We classified those species into 10 groups (dogs, foxes, and wolves belonging to the same canine family) by taxonomic family (Fig. S2B).

### Phylogenetic analysis and clustering

The likelihood mapping plot reveals the phylogenetic information of the alignment, with only 3.6% dots falling in the central area (representing unresolved quartets) and 92.5% dots falling at the corners of the triangle (representing fully resolved quartets), suggesting that there is sufficient phylogenetic information in the RABV full-length genome sequences (Fig. S12).

**Fig 1:**
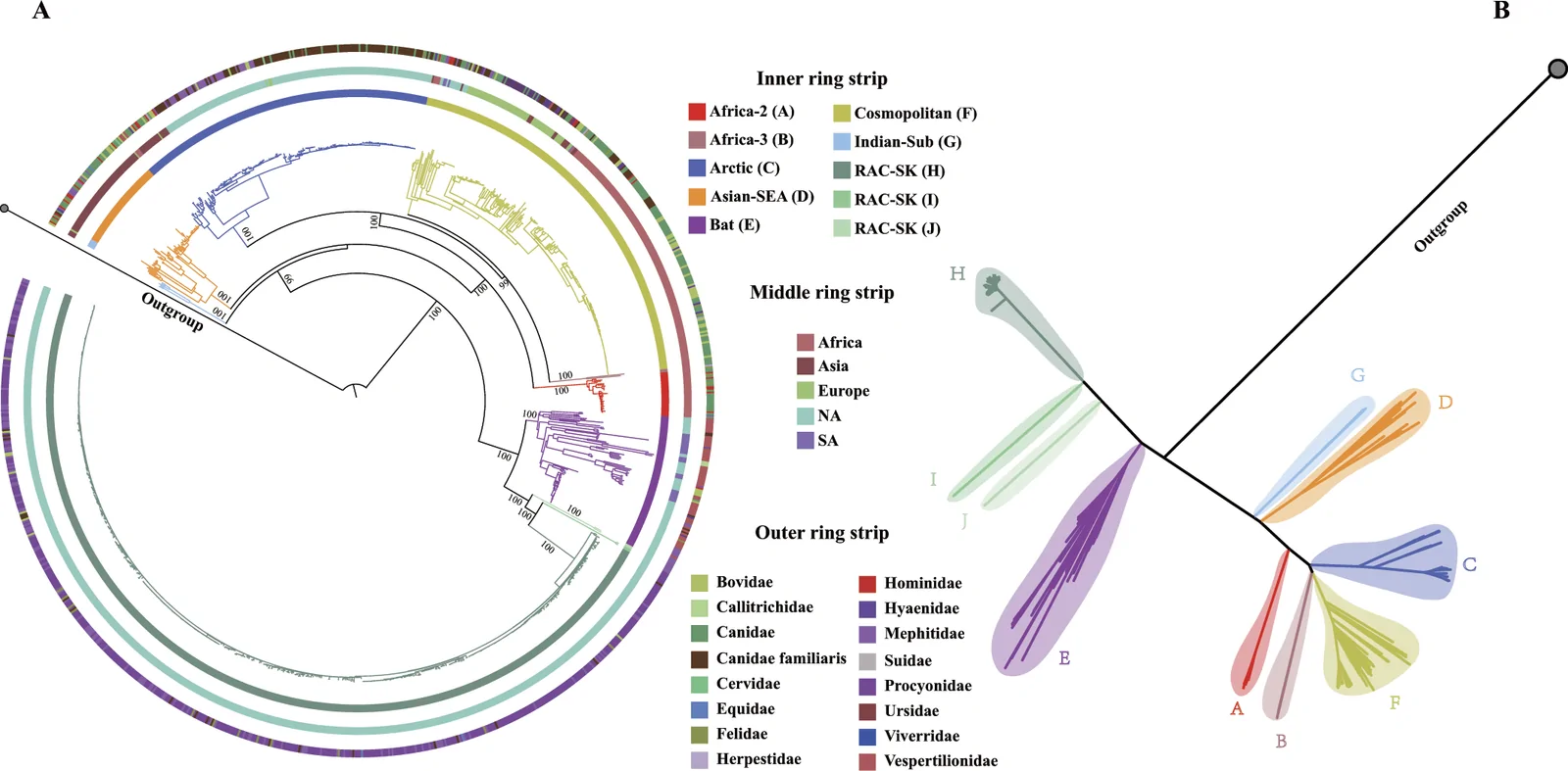
ML tree of full-length genomes. RAC-SK represents the group of American skunks and raccoons; SEA represents Southeast Asia; NA-M represents Middle North America, NA-N represents Northern North America; NA-S represents Southern North America; and SA represents South America. (**A**) Circular tree. Inner ring stripe represents the lineages in the previous study, middle ring stripe represents geographic distribution of RABV, and outer ring stripes represent different hosts of RABV. (**B**) Unroot tree.

The ML tree indicated that the genomes might be divided into 10 clades, roughly corresponding to the geographic distribution, in agreement with previous studies (Fig. 1A). In addition, in the RAC-SK clades, the three branches are far enough apart to suggest splitting them into three clades (H, I, and J), although there are few genomes in I and J clades (Fig. 1B). However, in terms of hosts, no significant distribution features were observed.

Subsequently, we conducted a principal component analysis to reduce the dimensions of the sequence information. Three principal components that best represent the sequence information were selected to determine genome clustering based on clades. The genomes were divided into 10 groups according to their clades, which is consistent with the results of the ML tree, although some boundaries are not very obvious in Arctic(C) and Cosmopolitan(F) clades (Fig. S3).

Both two analyses supported the division of the RABV genome into 10 major clades. Moreover, we ranked the host distribution, geographical distribution, and temporal distribution according to the clades (Table 1).

**TABLE 1:** The full-length genome distribution of rabies virus^*a*^

This study	Classics	Geographical distribution	Distribution of host families	Temporal distribution	Number
A	Africa-2	Africa	Felidae (2), Canidae (19), Hominidae (6), Mephitidae (1)	1986–2019	28
B	Africa-3	Africa	Felidae (1), Herpestidae (1)	2000	2
C	Arctic	NA-N (162), Europe (5), Asia (22)	Bovidae (9), Felidae (1), Cervidae (1), Canidae (135), Herpestidae (1), Hominidae (5), Mephitidae (36), Ursidae (1)	1977–2018	189
D	Asian-SEA	Asia	Bovidae (6), Felidae (1), Cervidae (1), Canidae (31), Equidae (1), Hominidae (9), Mephitidae (7), Suidae (1)	1956, 1983–2020	57
E	Bat	SA (25), NA-S (61)	Vespertilionidae (51), Bovidae (5), Felidae (1), Callithricidae (3), Canidae (11), Hominidae (1), Mephitidae (14)	1975–2020	86
F	Cosmopolitan	Asia (16), Africa (149), SA (3), NA (14), Europe (60)	Bovidae (27), Felidae (1), Cervidae (4), Canidae (193), Herpestidae (1), Hominidae (8), Hyaenidae (1), Mephitidae (36), Ursidae (1), Viverridae (1)	1931, 1950, 1973–2018	244
G	Indian-Sub	Asia (4), Europe (1)	Bovidae (1), Felidae (1), Canidae (1), Hominidae (2)	2008–2017	5
H	RAC-SK	NA-M	Bovidae (9), Felidae (12), Canidae (21), Hominidae (2), Equidae (2), Mephitidae (126), Procyonidae (421)	1989–2018	587
I		NA-M	Mephitidae (2)	2000s	2
J		NA-M	Mephitidae (2)	2000s	2

^*a*^ The figures in brackets represent the number of samples.

### Recombination analysis

We used RDP4 to assess the recombination events of the full-length genome sequences. Eight recombinant events, including intra- and inter-clade recombination, occurs in the sequences of Arctic(C), Asian-SEA(D), and Cosmopolitan(F) clades. Of note, all of these 14 recombinant genomes were collected from China and involved the families Bovidae, Canidae, Hominidae, and Mephitidae, suggesting viral recombinant might contribute to cross-species transmission (Table 2). One of the eight events occurred to the N gene, whereas the other six occurred to the L gene. There was a widespread and suspicious recombination from the N gene to the L gene in the eighth event (Fig. S4). Subsequently, GARD was applied to find evidence of recombination breakpoints, and 28 possible recombination breakpoints were found, of which three were in N gene and 20 in L gene with high support values (Fig. S5).

**TABLE 2:** Recombination of full-length genome detection by RDP4

No.	Recombination	Major parent	Minor parent	Detection methods						
				R^*a*^	G	B	M	C	S	T
A	EU643590	KY451767	JN234411	+^*b*^	+	−^*c*^	+	+	+	+
B	FJ712194	JQ970482	FJ712196	+	+	−	+	+	+	+
C	FJ712193	JQ970482	KF726852	+	+	−	+	+	+	+
D	FJ712194	FJ712193	FJ712195	+	+	+	+	+	+	+
E	FJ712195	FJ712196	JQ970482	+	+	+	+	+	+	+
F	FJ712195	KF726852	KC252634	+	+	+	+	+	+	+
G	FJ712195	KF726852	GU345748	+	+	+	+	+	−	+
H	KY997452	KY175229	KY912036	+	+	+	+	+	+	+

^*a*^ R represent RDP; G represent GENECONV; B represent Bootscan; M represent MaxChi; C represent Chimaera; S represent SisScan; T represent 3Seq.

^*b*^ “+” represents positive.

^*c*^ "−” represents negative.

### Temporal signal analysis

Although we initially assessed the temporal signals of the full-length genomes and structural protein genes in 1,202 RABV sequences, the small value of *R* ^2^ indicated that the time signals were weak (Fig. S6). In addition, according to the results of TreeTime, most sequences considerably deviated from the molecular clock model, and no significant signal was observed (Fig. S7). Therefore, the sequences were divided into five groups according to the ML tree, without Africa-2(A), Africa-3(B), and Indian-Sub(G). Based on the results, we determined that the N and L genes, with the high value of *R* ^2^ and suitable evolutionary rate and root age, could be used for BEAST analysis of RABV evolution and phylogeographic inference (Fig. S8). Moreover, the Bayesian evaluation of temporal signals (BETS) revealed that both the N and L genes with sampling dates were better than those without sampling dates (Table S2).

### Evolutionary analysis and phylogeographic inference

The best-fitting model was GTR + F + G4 with an uncorrelated relaxed clock and a coalescent Bayesian skyline for the N gene and TVM + F + G4 with a strict clock and a coalescent Bayesian skyline for the L gene. However, because the value of those effective sample sizes (ESSs) hardly exceeded 200, we selected the coalescent constant size as the tree prior for the N and L genes (Table S3).

**Fig 2:**
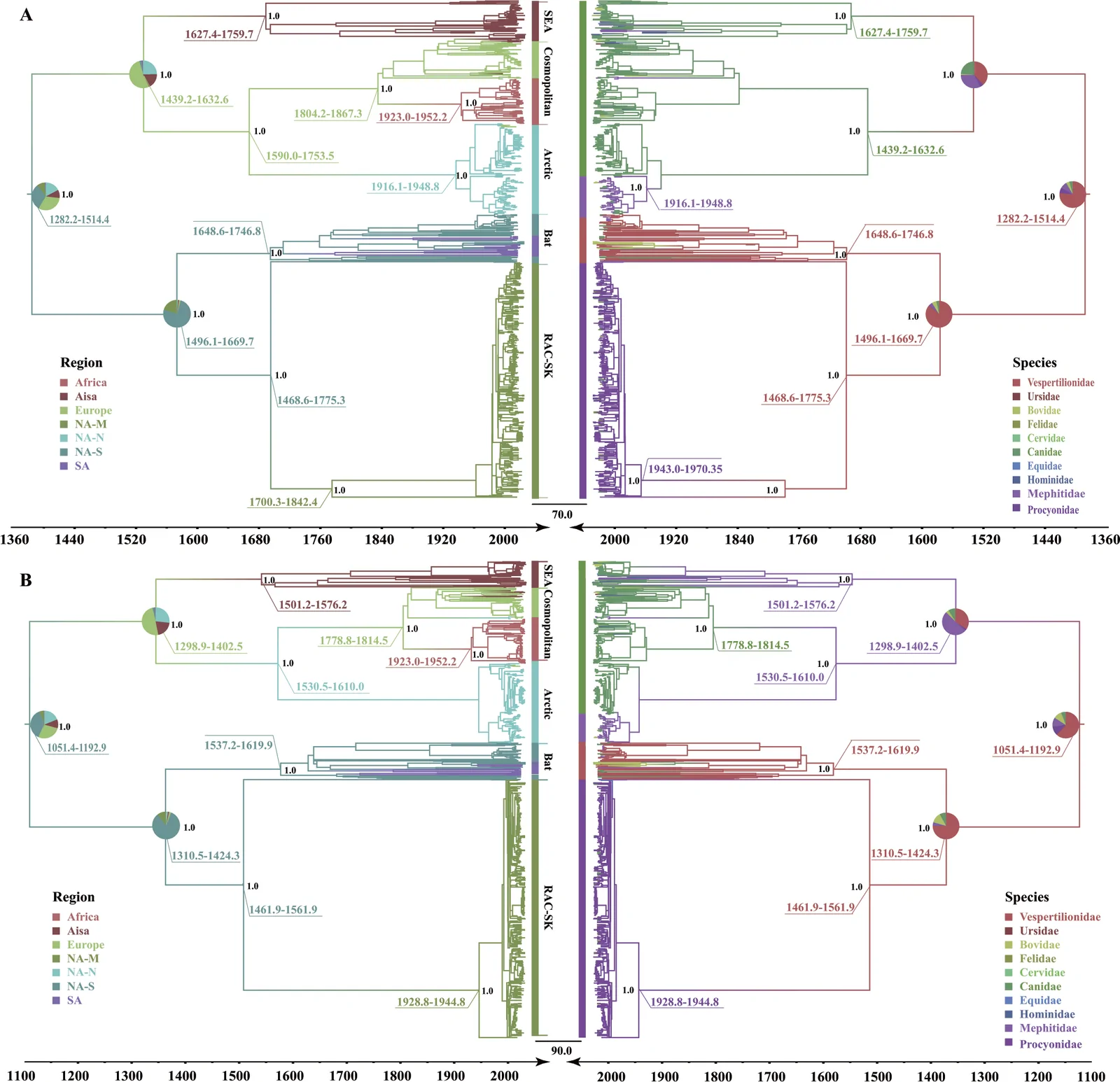
Phylogeographic reconstruction of N and L genes with different regions and hosts. Different colors represent different regions or different host families, the pie charts represent different probability values among regions or host families, the line colors represent the traits with the highest posterior probability value, and the numbers after the node represent posterior values. (**A**) MCC trees of N with traits of geographic distribution and different hosts. (**B**) MCC trees of L with traits of geographic distribution and different hosts.

Two MCC trees were reconstructed for the N and L genes, based on the region and host traits, which were visualized in FigTree. The analysis of both genes indicated that RABV most likely originated from bats (PP = 0.75 and PP = 0.60 inferred from N genes and L genes, separately) in North America (PP = 0.57 and PP = 0.62 inferred from N genes and L genes, separately). Due to the difference in the evolutionary rate of the N gene (2.22 × 10^−4^ subs/site/year, 95% HPD 1.99–2.47 × 10^−4^ subs/site/year) and L gene (1.67 × 10^−4^ subs/site/year, 95% HPD 1.59–1.74 × 10^−4^ subs/site/year), the root age was 1,406.6 (95% HPD 1282.2–1514.4) for the N genes and 1,122.7 (95% HPD 1051.4–1192.9) for the L genes. Subsequently, there are two major branches from the root. One branch (1,439.2–1,632.6, inferred from N gene; 1,298.9–1,402.5, inferred from L gene) is related to Canidae, including SEA(D), Cosmopolitan(F) and Arctic(C), and the other branch (1,496.1–1,669.7, inferred from N gene; 1,310.5–1,424.3, inferred from L gene) is related to Vespertilionidae and Musteloidea, including RAC-SK(H) and Bat(E). Then the dog-related branch was divided into three sub-clades, SEA(D) (1,627.4–1,759.7, N gene; 1,501.2–1,576.2, L gene), Cosmopolitan(F) (1,804.2–1,867.3, N gene; 1,778.8–1,814.5, L gene) and Arctic(C) (1,916.1–1,948.8, N gene; 1,923.0–1,952.2, L gene). Moreover, the other branch was divided into two sub-clades, Bat(E) (1,647.6–1,745.8, N gene; 1,527.2–1,619.9, L gene) and RAC-SK(H) (1,468.6–1,775.3, N gene; 1,461.9–1,561.9, L gene) (Fig. 2).

In terms of the host families, the Vespertilionidae group was their same ancestor. The Canidae group was primarily distributed in Africa, Asia, Europe, and NA-N, whereas the Mephitidae group was distributed throughout North America. There were the most Vespertilionidae species in NA-S and SA, while the Procyonidae group was unique to NA-M. The greatest difference between the N and L genes’ MMC trees was the transmission between the Vespertilionidae, Canidae, and Mephitidae groups. The transmission inferred from N genes might be from the Vespertilionidae group through the Canidae group to the Mephitidae group, whereas that from the L genes was from the Vespertilionidae group through the Mephitidae group to the Canidae group (Fig. 2).

**Fig 3:**
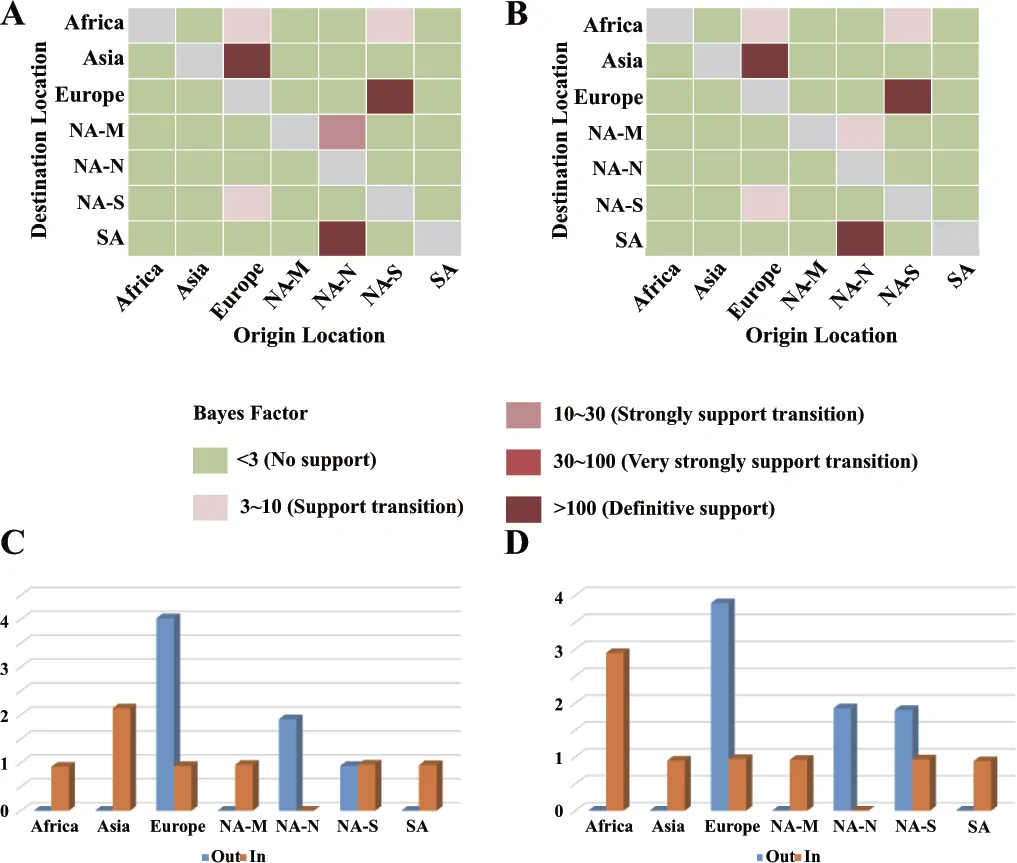
BF supports and migration rate for different regions. (**A**) Sufficient signal transmission routes inferred from N gene. (**B**) Sufficient signal transmission routes inferred from L gene. (**C**) The migration changes of RABV in each region inferred from N gene. (**D**) The migration changes of RABV in each region inferred from L gene.

**Fig 4:**
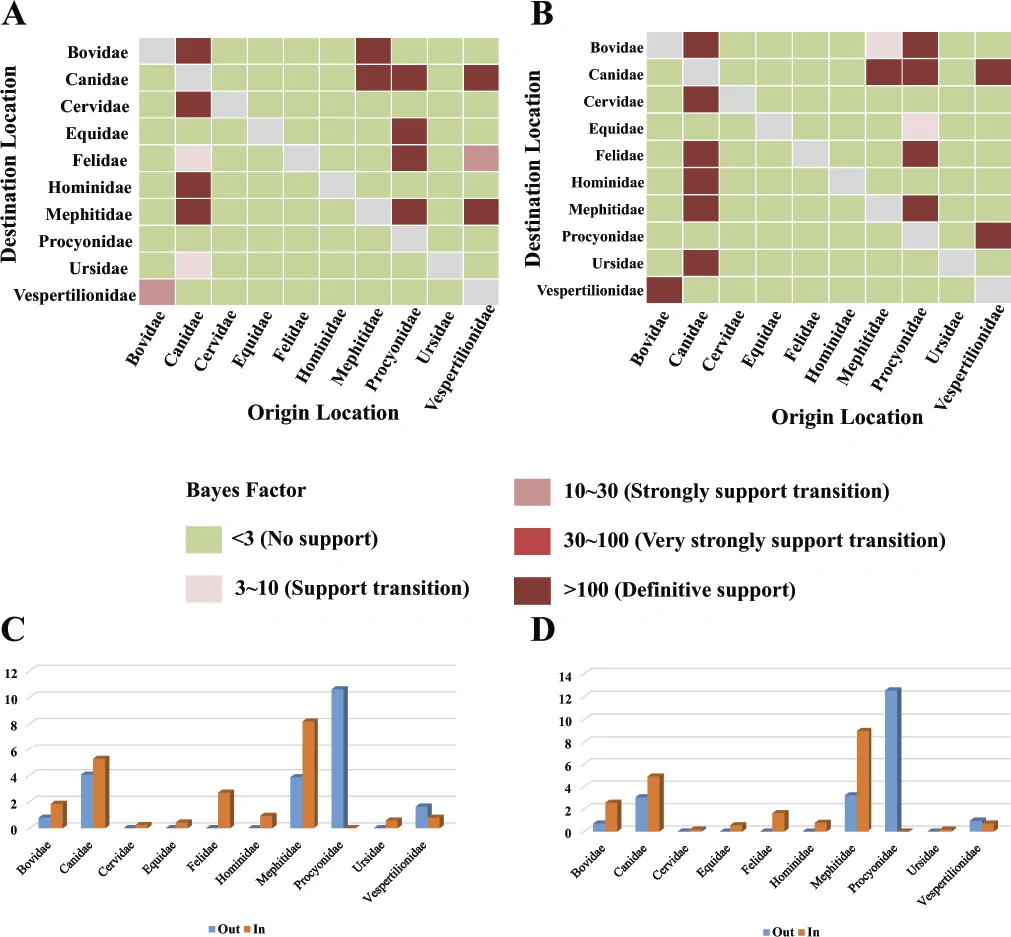
BF supports and migration rate of transmission for different hosts. (**A**) Sufficient signal transmission routes inferred from N gene. (**B**) Sufficient signal transmission routes inferred from L gene. (**C**) The migration changes of RABV in each region inferred from N gene. (**D**) The migration changes of RABV in each region inferred from L gene.

**Fig 5:**
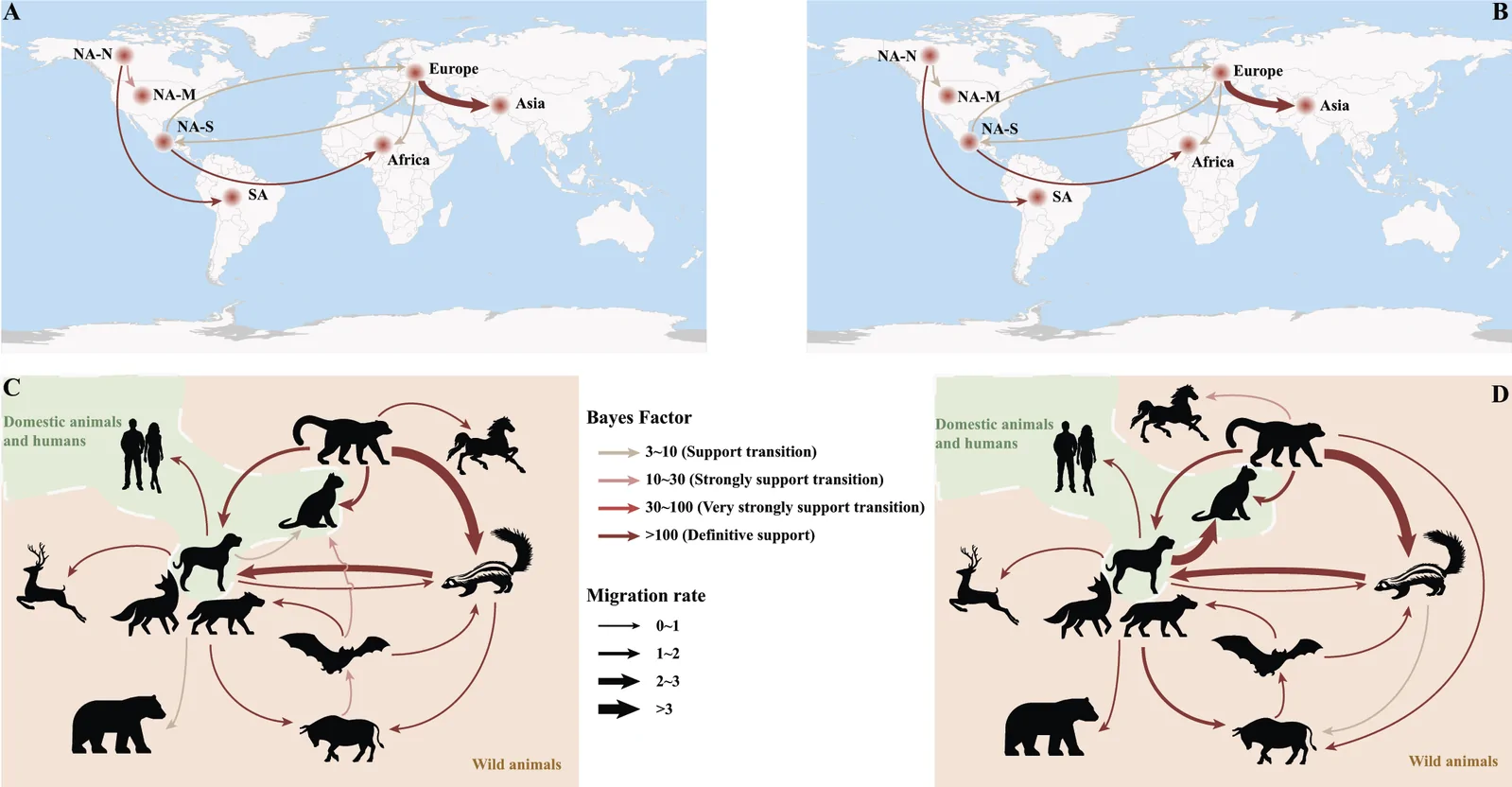
Spatiotemporal diffusion of RABV in different regions and hosts. Different colors of the arrow represent the Bayes Factor value; different widths of the arrow represent the migration rate. (**A**) The spatial diffusion inferred from N gene in different regions. (**B**) The spatial diffusion inferred from L gene in different regions. (**C**) The spatial diffusion inferred from N gene in different hosts. (**D**) The spatial diffusion inferred from L gene in different hosts.

The worldwide spatial dispersal networks of RABV based on the N and L genes were reconstructed. Concerning the N gene, there were seven discrete sampling locations and seven significant transmission routes for geographic distribution (Table S4). Europe, NA-N, and NA-S were the major outputs of RABV, and three transmission routes’ BF values exceeded 1,000—from Europe to Asia, from NA-S to Europe, and from NA-N to SA—indicating a strong correlation (Fig. 3A, C and 5A). Moreover, there were 10 discrete sampling host families and 16 significant transmission routes. The major output of RABV was from Procyonidae, Mephitidae, and Canidae (Table S5). Moreover, there were seven transmission routes with BF values over 1,000. The unique spread to Hominidae was via Canidae (Fig. 4A, C and 5B). Compared with the N genes, the L gene’s transmission routes in relation to the sampling locations were almost identical (Fig. 3B, D and 5C; Table S6). There were also 10 discrete sampling host families and 16 significant transmission routes (Table S7). Procyonidae, Mephitidae, and Canidae were the major outputs of RABV too. Similar to the N gene, the L gene’s unique spread to Hominidae was via Canidae, with high BF supports and posterior probability values (Fig. 4B, D and 5D). Those results suggested that the RABV unique spread to Hominidae could be via Canidae and that Mephitidae, Canidae played an important role in the transmission of RABV, both input and output.

We estimated the linear distance between each geographical distribution (https://map.baidu.com/) and the genetic distance between different families (https://www.onezoom.org/life.html) to determine their relationship with migration rate. The relationship between geographical distance and migration rate was not significant. Notably, a certain correlation between genetic distance and migration rate suggested that RABV tends to spread to the population with the shorter genetic distance from the host (Fig. S10).

### Genome-wide association studies (GWAS) and selection pressures analysis in different genes of different clades

**Fig 6:**
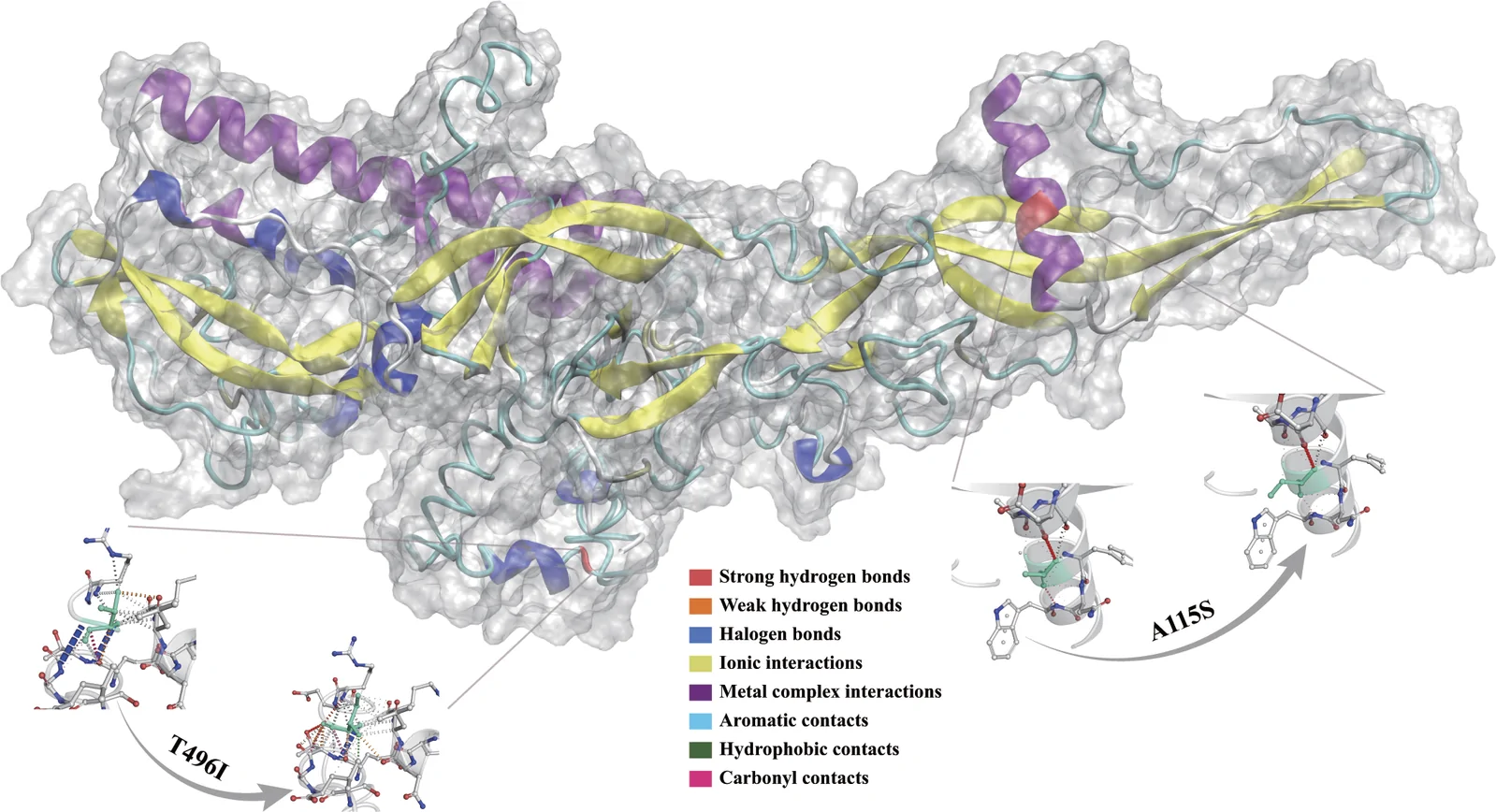
Location of selected amino acids in the structure of the G protein. It showed the structure of rabies virus G protein and the mutation of amino acid 115 from Ala to Ser and the mutation of amino acid 496 from Thr to Ile.

Since RABV was presumed to originate in bats and then spread to other animals, the full-length genome sequences were divided into three main lineages, bat-related, dog-related, and RAC-SK-related, to find key mutations for cross-species transmission. Using GWAS, we analyzed the traits of different RABV clades regarding their relationships with the SNVs of the RABV genome. Three SNV sites were identified at 3,325 (in the non-coding region), 3,660 (amino acid 115 of the G protein; G115) and 4,803 (G496)—with -lg (*P* values) over 7.3 (Fig. S11). We found there was a certain difference in these SNVs among the three lineages (Table 2). Bat-related and dog-related clades were consistent at sites 3,252 and 3,660, with most bases being c and g, whereas the RAC-SK-related clades were a and c, separately. Notably, at site 4,803, the dog-related and RAC-SK-related clades were identical, with most bases being a, whereas the bat-related clade was t. Because 3,660 and 4,803 SNVs are located on the G protein, we also compared the amino acid changes between these three lineages (Fig. 6). At the 3,660 (G115) and 4,803 (G496), different lineages showed different trends (Table 3).

**TABLE 3:** Detection of SNVs with significant differences by GWAS^*a*^

Lineages	Site				
	Nucleotide			Amino acids of G	
	3252	3660	4803	115	496
Bat-related (86)	c (80.2%), t (19.8%)	g (97.6%), t (1.2%)	t (95.3%), c (4.7%)	Ala (95.3%), Ser (4.7%)	Thr (83.7%), Ser (11.6%)
Dog-related (525)	c (99.8%), t (0.2%)	g (96.1%), t (2.4%)	a (95.0%), g (4.0%)	Ala (96.0%), Ser (2.4%)	Ile (61.9%), Thr (31.4%)
RAC-SK-related (591)	a (100%)	t (99.7%), a (0.3%)	a (98.6%), t (1.0%)	Ser (99.7%), Ala (0.3%)	Ile (85.3%), Met (13.1%)

^*a*^ The figures in brackets represent the number of samples or the percentage of total samples; a, t, g, and c represent nucleic acid base.

To determine whether the differences in evolutionary rates were due to different selective pressures, we classified the sequences by clade and evaluated the *d_N_/d_S_* values for each of the five genes. The values of N and L genes remain low and unfluctuating, with less selective pressure. Positive sites were identified as positively selected by at least three of these methods, MEME, FEL, FUBAR, and SLAC. A total of seven amino acid sites, positions 71, 172, and 174 in the P genes and position 212 in G genes for Arctic clade, position 496 in G genes for Bat clade, position 158 in P genes for Cosmopolitan clade, and position 883 in L genes for RAC-SK clade, were identified (Table 4) (27, 28). Of note, G496 was detected by both GWAS and selective pressure analysis, suggesting that it may be a key mutation that alters RABV host tropism under selection pressure (39, 40).

**TABLE 4:** Positive selected sites in five genes of rabies virus from different clades^*a*^

Clades	Gene	MEME	FEL	FUBAR	SLAC	*d_N_/d_S_* (by SLAC)
Africa-3	N	−	−	−	−	0.0228
	P	131, 143	−	−	−	0.114
	M	−	−	−	−	0.0813
	G	523	−	−	−	0.101
	L	118, 203, 303, 316, 328, 355, 491, 1,791, 1,804, 2,043	−	2,043	−	0.0518
Arctic	N	109, 126,128, 333	−	281	−	0.0619
	P	71, 162, 174	71, 172, 174	71, 160, 172, 174	172, 174	0.189
	M	88, 90 ,95	−	−	−	0.107
	G	42, 212	212	212	212	0.0995
	L	421, 426, 1,069, 1,070, 1,071, 1,076, 1,077, 1,226, 1,280, 1,327, 1,355, 1,356, 1,362, 1,369, 1,381, 1,392, 1,630, 1,632, 1,640, 1,641, 1,642, 1,643, 1,644, 1,647, 1,650	−	−	−	0.067
Bat	N	61, 385	−	−	−	0.0442
	P	−	135	−	−	0.147
	M	155	−	−	−	0.0579
	G	126, 127, 129, 212, 496	496	496	−	0.0922
	L	61, 203, 221, 335, 368, 726, 826, 1,138, 1,157, 1,618	335, 878, 1071	−	−	0.0457
Cosmopolitan	N	280, 283, 285, 290, 438	−	−	−	0.046
	P	149, 154, 158, 297	68, 158	158	158	0.153
	M	−	−	−	−	0.0904
	G	34, 84, 198, 212, 225, 266, 321, 470, 473	−	−	−	0.11
	L	98, 189, 888, 934, 940, 942, 944, 1,240, 1,249, 1,801, 2,034	−	−	−	0.0487
India	N	−	−	−	−	0.0167
	P	−	−	150	−	0.0655
	M	−	−	16	−	0.0624
	G	174, 184	490	490	−	0.66
	L	313, 314, 1,136	−	−	−	0.031
RAC-SK	N	98	98	−-	−	0.041
	P	195		−	−	0.158
	M	143	−	−	−	0.0913
	G	40, 318, 321,347, 367, 459, 474, 487, 490, 500	−	−	−	0.118
	L	80, 654, 883, 938, 1,008, 1,231, 1,804, 2,056	80, 883	883	883	0.0495
SEA	N	−	−	−	−	0.0359
	P	280	−	−	−	0.117
	M	−	−	−	−	0.0756
	G	72, 150	−	−	−	0.0863
	L	300, 363, 421, 430, 487, 847, 1,125, 1,425, 1,444, 1,484, 1,491,1,569, 1,636, 1,638, 1639, 1,641, 1,794, 1,810, 1,977, 2,086, 2,088, 2,094	−	−	−	0.0516

^*a*^ Codon positions with a *P*-value < 0.05 for the SLAC, FEL, and MEME models, and posterior of probability >0.95 for the FUBAR method were considered as containing evidence for positive selection. Screening positive selected sites that exist in more than three algorithms. The result does not include Africa-2 clade, due to only two sequences in it.

In terms of the mutation G115 from Ala to Ser, the ΔΔG = −0.748 kcal/mol and the ΔΔS_Vib ENCoM_= −0.90 kcal/mol/K indicating decreased stability and molecular flexibility of the G protein. Compared to Ala, the Ser increased spatial hindrance and alters the interaction between molecules. According to the value of ΔΔG (1.962 kcal/mol) and ΔΔS_Vib ENCoM_ (−1.128 kcal/mol/K), the stability of G protein was not reduced, but the flexibility was decreased at the mutant G496 from Thr to Ile. Compared to Ser, Ile changed the spatial position of the carbonyl group to form hydrogen bonds with more atoms and also adds a hydrophobic contact. Meanwhile, it was predicted by NetPhos that Ser of G496 might be the phosphorylation site and mutation would lead to the loss of phosphorylation function (Fig. 6) (41, 42).

## DISCUSSION

For more than 4,000 yr, rabies has plagued almost every corner of the world and has repeatedly crossed the host barrier between different species, which continues to pose a serious threat to animal safety and public health worldwide (43). Here, we adopted a phylogeographic approach to offer a panorama view about origin, evolution, and potential transmission routes of RABV. We revealed phylogenetically distinct clades co-circulating globally, together representing 10 families of host infected with RABV in 10 clades worldwide. In light of the stigma of coronavirus and monkeypox virus, we propose to change the traditional RABV clades nomenclature to reduce unnecessary misunderstandings and conflicts, and we renamed the clades with letters (44, 45). Moreover, we found that multiple inter- and intra-clade recombination events frequently occurred in the genomes collected from China. Viral recombination has the potential to alter the spreading ability of viruses, leading to unknown and potentially dangerous spillover risks. Recombination events between canine coronavirus (CCoV) and feline coronavirus (FCoV) have resulted in the formation of CCoV-HuPn-2018 and HuCCoV_Z19Haiti, which have the ability to cross species and infect humans, thereby becoming the ninth coronavirus known to infect humans (46 – 48). Therefore, it is imperative that we pay close attention to these recombinant events and their associated sequences.

Our study introduces L genes for the first time to reconstruct the spatiotemporal diffusion network of rabies virus. Previous studies have primarily focused on dog-related RABV and utilized the N gene for analysis (7, 8). Cecile et al. combined the N, P, M, G, and L genes to analyze the evolutionary history of dog-related RABV and established the currently accepted nomenclature for the major viral clades. The results showed an evolutionary rate and root age of 2.10–2.80 × 10^–4^ subs/site/year (95% HPD) and 1,308–1,510 (95% HPD), respectively, which is consistent with our findings from the L gene analysis (7, 8). Additionally, we combined the L gene sequences of bat-related RABV, dog-related RABV, and RAC-SK-related RABV and gain a stronger temporal signal and a more accurate assessment of time and evolutionary rate with a narrower confidence interval. Our comprehensive analysis reveals the diversity, host distribution, genetic evolution, origin, and spatial and temporal distribution of the rabies virus. Moreover, in this study, we isolated a strain of RABV from infected pigs that could be stably passed along the NA and BHK cell lines (49). Due to the RABV repeatedly crossing the host barrier between different animals, our study thus amounted to the first comprehensive phylogeographic exploration of RABV among susceptible animal species in different families around the world. Analyzing the N and L genes using the Bayesian stochastic search variable selection traits model revealed that the origin, evolution, and potential transmission routes were similar, which suggests that RABV may originate from bats in North America. Europe has the most involved transmission routes, possibly due to the colonial expansion of European countries that began in the 15th century. Therefore, we speculate that rabies virus originated in North America and spread throughout the world via European colonial expansion and developed maritime capabilities over the course of three centuries. According to the phylogeographic inference, Mephitidae might be the intermediate host of rabies from Vespertilionidae to Canidae. Among other results, there was a certain correlation between genetic distance and migration rate, which implies that RABV has tended to spread to populations with a short genetic distance from the host. On that note, in terms of genetic distance, Mephitidae is closer to Vespertilionidae than Canidae and may thus be an ideal intermediate host.

To analyze the effect of gene mutation, we used GWAS and positive selection to identify three SNVs and seven positive selected sites, which may impact the rabies virus. We reconstructed 3D models of G proteins based on mutations at sites 115 and 496 and compared the changes to explore the effects of these mutations on protein structure and function. At the genetic level, positive selection of amino acids in the functional regions of the virus plays a role in the host transfer of RABV (50, 51). The glycoprotein of RABV is a surface protein of the virus and a key protein for recognizing host receptors. Therefore, it deserves significant attention. Position 496 in the glycoprotein was identified as a potential phosphorylation site and was selected by GWAS and positive selection. The G496 mutant from Thr to Ile changed the spatial position of the carbonyl group to form hydrogen bonds with more atoms and also adds a hydrophobic contact, which would lead to the loss of phosphorylation function, which might alter the host tropism of rabies (39, 40).

The study had several limitations. In terms of sampling bias, the information from sequences does not represent all regions because some places showed infections with RABV but had no sequence uploaded in GenBank. We sought to mitigate sampling bias by deleting sequences based on fewer sequence groups. However, doing so considerably depleted the amount of useful information available for our analysis and made the results incomprehensive. Therefore, we deleted some clades or host families groups with few sequences and used five major clades to reduce sampling bias (27, 52).

### Conclusions

Our findings provide a panoramic view of the evolution and distribution of rabies virus. In addition, we briefly analyzed important amino acid mutations that may lead to cross-species transmission of RABV. There is growing evidence that the risk of RABV spillover is increasing. Therefore, comprehensive surveillance and enhanced biosecurity precautions should be taken to achieve the goal of canine-mediated rabies eradication.

## SUPPLEMENTAL MATERIAL

Supplemental Material: Tables and Figures spectrum.05257-22-s0001.docx Supplemental Material: Tables S1-S7 and Figures S1-S12

ASM does not own the copyrights to Supplemental Material that may be linked to, or accessed through, an article. The authors have granted ASM a non-exclusive, world-wide license to publish the Supplemental Material files. Please contact the corresponding author directly for reuse.
