# Supplementary material for: Evolution and distribution of rabies viruses from a panorama view
Source: Microbiol Spectr. 2023 Sep 5;11(5):e05257-22. doi: 10.1128/spectrum.05257-22 (PMC10581214; doi:10.1128/spectrum.05257-22)
Supplement: Supplemental Material: Tables and Figures — Supplemental Material: Tables S1-S7 and Figures S1-S12 [file spectrum.05257-22-s0001.docx]

**Supplemental Material: Tables and Figures**

**Table S1 RABV sequences information**

| **Accession** | **Country** | **Collection Date** | **Host** |
| --- | --- | --- | --- |
| AB569299 | Sri Lanka | 2008 | Homo sapiens |
| AB635373 | Sri Lanka | 2009 | Paradoxurus zeylonensis |
| AB981663 | Laos | 2011 | Canis lupus |
| AB981664 | Laos | 2011 | Canis lupus |
| EF564174 | China | 1956 | Homo sapiens |
| EU293111 | Thailand | 1983 | Homo sapiens |
| EU293115 | France | 1991 | Canidae |
| EU293116 | Argentina | 1997 | Tadarida brasiliensis |
| EU643590 | China | 2006 | Homo sapiens |
| FJ712193 | China | 2008 | Canis lupus familiaris |
| FJ712194 | China | 2008 | Canis lupus familiaris |
| FJ712195 | China | 2008 | Melogale moschata |
| FJ712196 | China | 2008 | Melogale moschata |
| FJ866835 | China | 2008 | Canis lupus familiaris |
| FJ866836 | China | 2008 | Canis lupus familiaris |
| GQ412744 | China | 1931 | Canis lupus familiaris |
| GU345746 | China | 1992 | Canis lupus familiaris |
| GU345747 | China | 1986 | Homo sapiens |
| GU345748 | China | 2006 | Canis lupus familiaris |
| GU358653 | China | 1994 | Canis lupus familiaris |
| GU647092 | China | 2008 | Melogale moschata |
| HE802675 | Pakistan | 2010 | Bos taurus |
| HE802676 | Pakistan | 2007 | Bos taurus |
| HQ317918 | China | 1956 | Homo sapiens |
| HQ450385 | China | 2008 | Canis lupus familiaris |
| JN234411 | China | 1931 | Canis lupus familiaris |
| JN609295 | China | 2008 | Canis lupus familiaris |
| JQ647510 | China | 2011 | Equus asinus |
| JQ685893 | USA | 2001 | Mephitis mephitis |
| JQ685894 | USA | 1994 | Mephitis mephitis |
| JQ685895 | USA | 2003 | Lasionycteris noctivagans |
| JQ685897 | USA | 2001 | Eptesicus fuscus |
| JQ685898 | USA | 2009 | Eptesicus fuscus |
| JQ685899 | USA | 2009 | Urocyon cinereoargenteus |
| JQ685900 | USA | 2003 | Lasiurus seminolus |
| JQ685901 | USA | 2003 | Procyon lotor |
| JQ685902 | USA | 2005 | Lasiurus borealis |
| JQ685903 | USA | 2004 | Eptesicus fuscus |
| JQ685904 | USA | 2001 | Mephitis mephitis |
| JQ685905 | USA | 2003 | Tadarida brasiliensis |
| JQ685907 | USA | 1996 | Eptesicus fuscus |
| JQ685909 | USA | 2005 | Eptesicus fuscus |
| JQ685910 | USA | 2002 | Dasypterus xanthinus |
| JQ685911 | USA | 2001 | Mephitis mephitis |
| JQ685913 | USA | 1985 | Eptesicus fuscus |
| JQ685915 | USA | 2002 | Dasypterus intermedius |
| JQ685916 | USA | 2002 | Dasypterus intermedius |
| JQ685917 | USA | 2010 | Canis latrans |
| JQ685918 | USA | 2010 | Urocyon cinereoargenteus |
| JQ685919 | USA | 2005 | Lasiurus borealis |
| JQ685920 | USA | 1984 | Eptesicus fuscus |
| JQ685921 | USA | 2001 | Myotis austroriparius |
| JQ685922 | USA | 2005 | Perimyotis subflavus |
| JQ685923 | USA | 1999 | Eptesicus fuscus |
| JQ685925 | USA | 2004 | Eptesicus fuscus |
| JQ685929 | Mexico | 2009 | Spilogale putorius |
| JQ685931 | USA | 2000 | Eptesicus fuscus |
| JQ685932 | USA | 2001 | Mephitis mephitis |
| JQ685933 | USA | 2004 | Urocyon cinereoargenteus |
| JQ685934 | USA | 2009 | Urocyon cinereoargenteus |
| JQ685938 | USA | 2009 | Mephitis mephitis |
| JQ685940 | USA | 2001 | Mephitis mephitis |
| JQ685941 | USA | 2005 | Mephitis mephitis |
| JQ685942 | USA | 1981 | Eptesicus fuscus |
| JQ685943 | USA | 2009 | Urocyon cinereoargenteus |
| JQ685944 | USA | 1984 | Mephitis mephitis |
| JQ685945 | USA | 2005 | Felis catus |
| JQ685946 | USA | 1999 | Eptesicus fuscus |
| JQ685947 | USA | 2004 | Aeorestes cinereus |
| JQ685948 | USA | 2010 | Urocyon cinereoargenteus |
| JQ685950 | USA | 2009 | Eptesicus fuscus |
| JQ685952 | USA | 2002 | Parastrellus hesperus |
| JQ685953 | Mexico | 2009 | Homo sapiens |
| JQ685954 | Mexico | 2007 | Spilogale putorius |
| JQ685955 | USA | 2005 | Myotis yumanensis |
| JQ685956 | USA | 1975 | Eptesicus fuscus |
| JQ685957 | USA | 2009 | Urocyon cinereoargenteus |
| JQ685958 | USA | 2001 | Mephitis mephitis |
| JQ685959 | USA | 2001 | Mephitis mephitis |
| JQ685960 | USA | 2001 | Eptesicus fuscus |
| JQ685961 | USA | 2010 | Eptesicus fuscus |
| JQ685962 | USA | 2001 | Mephitis mephitis |
| JQ685963 | Mexico | 2009 | Nasua narica |
| JQ685964 | USA | 2004 | Mephitis mephitis |
| JQ685965 | USA | 2002 | Parastrellus hesperus |
| JQ685966 | USA | 2001 | Mephitis mephitis |
| JQ685968 | USA | 2009 | Mephitis mephitis |
| JQ685969 | USA | 2001 | Mephitis mephitis |
| JQ685970 | USA | 1974 | Mephitis mephitis |
| JQ685971 | USA | 2009 | Antrozous pallidus |
| JQ685973 | USA | 2011 | Canis latrans |
| JQ685974 | USA | 1995 | Eptesicus fuscus |
| JQ685975 | Mexico | 2009 | Spilogale putorius |
| JQ730682 | China | 2010 | Canis lupus familiaris |
| JQ944704 | Russia | 2009 | Nyctereutes procyonoides |
| JQ944705 | Russia | 2008 | Canis lupus familiaris |
| JQ944706 | Russia | 2008 | Canis lupus familiaris |
| JQ944707 | Russia | 2008 | Cervidae |
| JQ944708 | Russia | 2008 | Vulpes vulpes |
| JQ970480 | China | 2008 | Canis lupus familiaris |
| JQ970481 | China | 2006 | Canis lupus familiaris |
| JQ970482 | China | 2006 | Canis lupus familiaris |
| JQ970483 | China | 2008 | Canis lupus familiaris |
| JQ970484 | China | 2009 | Canis lupus familiaris |
| JQ970485 | China | 2009 | Canis lupus familiaris |
| JQ970486 | China | 2007 | Canis lupus familiaris |
| JQ970487 | China | 2009 | Canis lupus familiaris |
| JX088694 | China | 2012 | Pig |
| JX473838 | Namibia | 2009 | Canis |
| JX473839 | Namibia | 2009 | Canis |
| JX473840 | Namibia | 2009 | Tragelaphus strepsiceros |
| JX473841 | Namibia | 2009 | Tragelaphus strepsiceros |
| KC169986 | China | 2009 | Bos taurus |
| KC171643 | South Korea | 2008 | Bos taurus |
| KC171644 | South Korea | 2004 | Nyctereutes procyonoides |
| KC171645 | South Korea | 1999 | Nyctereutes procyonoides |
| KC193267 | China | 2011 | Bos taurus |
| KC196743 | Nigeria | 2011 | Canis lupus familiaris |
| KC252633 | China | 2011 | Bos taurus |
| KC252634 | China | 2011 | Canis lupus familiaris |
| KC737850 | USA | 2011 | Homo sapiens |
| KC762941 | China | 2009 | Melogale moschata |
| KC977995 | China | 2009 | Canis lupus familiaris |
| KF154996 | UK | 1987 | Homo sapiens |
| KF154998 | Israel | 1950 | Canis lupus familiaris |
| KF154999 | UK | 2008 | Canis lupus familiaris |
| KF155000 | Iraq | 2010 | Bos taurus |
| KF155001 | Morocco | 2009 | Bos taurus |
| KF155002 | Tanzania | 2010 | Canis lupus familiaris |
| KF620487 | Taiwan | 2012 | Melogale |
| KF726852 | China | 2009 | Melogale moschata |
| KF726853 | China | 2010 | Melogale moschata |
| KF977826 | CAR | 2011 | Homo sapiens |
| KJ004416 | China | 2013 | Homo sapiens |
| KJ564280 | China | 2013 | Dama dama |
| KM016899 | China | 2014 | Vulpes vulpes |
| KM272192 | China | 2012 | Canis lupus familiaris |
| KM594023 | Brazil | 2008 | Callithrix jacchus |
| KM594024 | Brazil | 2012 | Callithrix jacchus |
| KM594025 | Brazil | 2007 | Callithrix jacchus |
| KM594026 | Brazil | 2009 | Eptesicus furinalis |
| KM594027 | Brazil | 2010 | Eptesicus furinalis |
| KM594028 | Brazil | 2010 | Eptesicus furinalis |
| KM594029 | Brazil | 2006 | Eptesicus furinalis |
| KM594030 | Brazil | 2010 | Myotis nigricans |
| KM594031 | Brazil | 2010 | Myotis nigricans |
| KM594032 | Brazil | 2010 | Myotis nigricans |
| KM594033 | Brazil | 2010 | Tadarida brasiliensis |
| KM594034 | Brazil | 2010 | Nyctinomops laticaudatus |
| KM594035 | Brazil | 2010 | Nyctinomops laticaudatus |
| KM594036 | Brazil | 2010 | Nyctinomops laticaudatus |
| KM594037 | Brazil | 2009 | Tadarida brasiliensis |
| KM594038 | Brazil | 2010 | Tadarida brasiliensis |
| KM594039 | Brazil | 2009 | Cerdocyon thous |
| KM594040 | Brazil | 2013 | Desmodus rotundus |
| KM594041 | Brazil | 2013 | Desmodus rotundus |
| KM594042 | Brazil | 2013 | Desmodus rotundus |
| KM594043 | Brazil | 2012 | Bos taurus |
| KP723638 | Ethiopia | 2014 | Canis simensis |
| KP997032 | Russia | 2014 | Ursus arctos |
| KR230089 | China | 2015 | Bos taurus |
| KR230090 | China | 2015 | Bos taurus |
| KR534217 | Tanzania | 2008 | Canis lupus familiaris |
| KR534218 | Tanzania | 2008 | Canis lupus familiaris |
| KR534219 | Tanzania | 2007 | Canis lupus familiaris |
| KR534220 | Tanzania | 2008 | Canis lupus familiaris |
| KR534232 | Tanzania | 2011 | Canis |
| KR534233 | Tanzania | 2011 | Canis lupus familiaris |
| KR534234 | Tanzania | 2011 | Bos taurus |
| KR534235 | Tanzania | 2011 | Bos taurus |
| KR534236 | Tanzania | 2011 | Bos taurus |
| KR534237 | Tanzania | 2011 | Bos taurus |
| KR534244 | Tanzania | 2011 | Canis lupus familiaris |
| KR534245 | Tanzania | 2011 | Bos taurus |
| KR534246 | Tanzania | 2011 | Viverridae |
| KR534247 | Tanzania | 2011 | Canis lupus familiaris |
| KR534248 | Tanzania | 2011 | Bos taurus |
| KR534250 | Tanzania | 2011 | Canis lupus familiaris |
| KR534251 | Tanzania | 2011 | Canis lupus familiaris |
| KR534252 | Tanzania | 2011 | Canis lupus familiaris |
| KR534254 | Tanzania | 2011 | Felis catus |
| KT006769 | Mexico | 2013 | Canis lupus familiaris |
| KT336432 | South Africa | 2012 | Canis lupus familiaris |
| KT336433 | Zimbabwe | 1991 | Canis lupus familiaris |
| KT336434 | Zimbabwe | 1992 | Canis lupus familiaris |
| KT336435 | Zimbabwe | 1993 | Canis lupus familiaris |
| KT336436 | South Africa | 2012 | Canis lupus familiaris |
| KT336437 | South Africa | 2012 | Canis lupus familiaris |
| KT728348 | Russia | 2003 | Homo sapiens |
| KT728349 | Russia | 2003 | Homo sapiens |
| KU198460 | USA | 1989 | Vulpes lagopus |
| KU198461 | Canada | 1990 | Vulpes lagopus |
| KU198462 | USA | 1990 | Vulpes lagopus |
| KU198463 | USA | 1990 | Vulpes vulpes |
| KU198464 | Canada | 1991 | Vulpes lagopus |
| KU198465 | Canada | 1992 | Vulpes vulpes |
| KU198466 | Canada | 1993 | Vulpes lagopus |
| KU198467 | Canada | 1997 | Vulpes lagopus |
| KU198468 | Canada | 2000 | Vulpes vulpes |
| KU198469 | Canada | 2001 | Vulpes vulpes |
| KU198470 | Canada | 2002 | Vulpes vulpes |
| KU198471 | USA | 2007 | Vulpes vulpes |
| KU198472 | Canada | 2012 | Vulpes lagopus |
| KU198473 | Canada | 2012 | Vulpes vulpes |
| KU198474 | Canada | 2012 | Vulpes vulpes |
| KU198475 | Canada | 2013 | Vulpes lagopus |
| KU198476 | Canada | 2013 | Vulpes lagopus |
| KU198478 | Canada | 2013 | Vulpes lagopus |
| KU198479 | Canada | 2014 | Vulpes vulpes |
| KU523255 | French Guiana | 2010 | Desmodus rotundus |
| KX036361 | Greenland | 1990 | Vulpes lagopus |
| KX036362 | Greenland | 2001 | Vulpes lagopus |
| KX036363 | Greenland | 2002 | Vulpes lagopus |
| KX036364 | Greenland | 2002 | Vulpes lagopus |
| KX036365 | Greenland | 2002 | Vulpes lagopus |
| KX036366 | Greenland | 2002 | Vulpes lagopus |
| KX036367 | Greenland | 2002 | Vulpes lagopus |
| KX148100 | French Guiana | 2009 | Stenodermatinae |
| KX148101 | Egypt | 1979 | Homo sapiens |
| KX148102 | Mexico | 1991 | Canis lupus familiaris |
| KX148103 | South Africa | 1981 | Homo sapiens |
| KX148104 | Montenegro | 1978 | Bos taurus |
| KX148105 | Greenland | 1980 | Canis lupus familiaris |
| KX148106 | USA | 1990 | Canidae |
| KX148107 | Benin | 1986 | Felis catus |
| KX148108 | Nepal | 2011 | Bos taurus |
| KX148109 | Brazil | 1986 | Canis lupus familiaris |
| KY026414 | USA | 2013 | Procyon lotor |
| KY026415 | Canada | 2014 | Mephitidae |
| KY026416 | USA | 1995 | Procyon lotor |
| KY026417 | USA | 1998 | Procyon lotor |
| KY026418 | USA | 1998 | Mephitidae |
| KY026419 | USA | 2004 | Mephitidae |
| KY026420 | USA | 2004 | Procyon lotor |
| KY026421 | USA | 2011 | Procyon lotor |
| KY026422 | Canada | 1999 | Procyon lotor |
| KY026423 | Canada | 1999 | Procyon lotor |
| KY026425 | Canada | 1999 | Procyon lotor |
| KY026426 | Canada | 2000 | Procyon lotor |
| KY026427 | Canada | 2000 | Procyon lotor |
| KY026428 | Canada | 2000 | Procyon lotor |
| KY026430 | Canada | 2000 | Procyon lotor |
| KY026431 | Canada | 2000 | Procyon lotor |
| KY026432 | Canada | 2000 | Procyon lotor |
| KY026433 | Canada | 2000 | Procyon lotor |
| KY026434 | Canada | 2000 | Procyon lotor |
| KY026435 | Canada | 2000 | Procyon lotor |
| KY026436 | Canada | 2000 | Procyon lotor |
| KY026437 | Canada | 2000 | Procyon lotor |
| KY026438 | Canada | 2000 | Procyon lotor |
| KY026439 | Canada | 2000 | Procyon lotor |
| KY026440 | Canada | 2000 | Procyon lotor |
| KY026442 | Canada | 2000 | Procyon lotor |
| KY026443 | Canada | 2000 | Procyon lotor |
| KY026444 | Canada | 2001 | Procyon lotor |
| KY026446 | Canada | 2001 | Procyon lotor |
| KY026447 | Canada | 2001 | Procyon lotor |
| KY026448 | Canada | 2001 | Procyon lotor |
| KY026449 | Canada | 2001 | Procyon lotor |
| KY026450 | Canada | 2001 | Mephitidae |
| KY026451 | Canada | 2001 | Procyon lotor |
| KY026452 | Canada | 2001 | Procyon lotor |
| KY026453 | Canada | 2001 | Procyon lotor |
| KY026454 | Canada | 2001 | Procyon lotor |
| KY026455 | Canada | 2001 | Procyon lotor |
| KY026456 | Canada | 2001 | Procyon lotor |
| KY026457 | Canada | 2001 | Procyon lotor |
| KY026458 | Canada | 2001 | Procyon lotor |
| KY026459 | Canada | 2002 | Procyon lotor |
| KY026460 | Canada | 2002 | Procyon lotor |
| KY026461 | Canada | 2002 | Procyon lotor |
| KY026462 | Canada | 2002 | Procyon lotor |
| KY026463 | Canada | 2002 | Procyon lotor |
| KY026464 | Canada | 2003 | Procyon lotor |
| KY026465 | Canada | 2003 | Procyon lotor |
| KY026466 | Canada | 2003 | Procyon lotor |
| KY026467 | Canada | 2003 | Procyon lotor |
| KY026468 | Canada | 2003 | Procyon lotor |
| KY026469 | Canada | 2003 | Procyon lotor |
| KY026470 | Canada | 2003 | Procyon lotor |
| KY026471 | Canada | 2003 | Procyon lotor |
| KY026472 | Canada | 2003 | Procyon lotor |
| KY026473 | Canada | 2004 | Procyon lotor |
| KY026474 | Canada | 2004 | Procyon lotor |
| KY026475 | Canada | 2004 | Procyon lotor |
| KY026476 | Canada | 2004 | Procyon lotor |
| KY026479 | USA | 2006 | Mephitidae |
| KY026480 | USA | 2007 | Procyon lotor |
| KY026481 | USA | 2008 | Procyon lotor |
| KY026482 | USA | 2008 | Procyon lotor |
| KY026483 | USA | 2011 | Mephitidae |
| KY175229 | China | 2012 | Canis lupus familiaris |
| KY175230 | China | 2015 | Homo sapiens |
| KY210220 | Tanzania | 2008 | Capra hircus |
| KY210222 | Tanzania | 2009 | Canis lupus familiaris |
| KY210223 | Tanzania | 2007 | Canis lupus familiaris |
| KY210224 | Tanzania | 2010 | Canis lupus familiaris |
| KY210225 | Tanzania | 2011 | Capra hircus |
| KY210227 | Tanzania | 2011 | Canis lupus familiaris |
| KY210229 | Tanzania | 2011 | Canis lupus familiaris |
| KY210230 | Tanzania | 2011 | Bos taurus |
| KY210231 | Tanzania | 2011 | Canis lupus familiaris |
| KY210232 | Tanzania | 2011 | Canis lupus familiaris |
| KY210233 | Tanzania | 2011 | Capra hircus |
| KY210235 | Tanzania | 2011 | Canis lupus familiaris |
| KY210236 | Tanzania | 2011 | Bos taurus |
| KY210237 | Tanzania | 2011 | Bos taurus |
| KY210238 | Tanzania | 2011 | Canis lupus familiaris |
| KY210239 | Tanzania | 2011 | Canis lupus familiaris |
| KY210240 | Tanzania | 2011 | Bos taurus |
| KY210241 | Tanzania | 2011 | Capra hircus |
| KY210243 | Tanzania | 2011 | Canis lupus familiaris |
| KY210244 | Tanzania | 2011 | Canis lupus familiaris |
| KY210245 | Tanzania | 2012 | Canis lupus familiaris |
| KY210246 | Tanzania | 2012 | Ovis aries |
| KY210247 | Tanzania | 2012 | Canis lupus familiaris |
| KY210248 | Tanzania | 2012 | Canis lupus familiaris |
| KY210249 | Tanzania | 2012 | Canis lupus familiaris |
| KY210250 | Tanzania | 2012 | Canis lupus familiaris |
| KY210251 | Tanzania | 2012 | Canis lupus familiaris |
| KY210252 | Tanzania | 2012 | Canis lupus familiaris |
| KY210253 | Tanzania | 2013 | Canis lupus familiaris |
| KY210254 | Tanzania | 2013 | Canis lupus familiaris |
| KY210255 | Tanzania | 2013 | Canis lupus familiaris |
| KY210256 | Tanzania | 2013 | Canis lupus familiaris |
| KY210258 | Tanzania | 2013 | Canis lupus familiaris |
| KY210259 | Tanzania | 2013 | Felis catus |
| KY210261 | Tanzania | 2013 | Capra hircus |
| KY210262 | Tanzania | 2013 | Capra hircus |
| KY210263 | Tanzania | 2013 | Canis lupus familiaris |
| KY210264 | Tanzania | 2013 | Canis lupus familiaris |
| KY210265 | Tanzania | 2013 | Canis lupus familiaris |
| KY210266 | Tanzania | 2013 | Canis lupus familiaris |
| KY210267 | Tanzania | 2013 | Canis lupus familiaris |
| KY210268 | Tanzania | 2013 | Bos taurus |
| KY210269 | Tanzania | 2013 | Canis lupus familiaris |
| KY210271 | Tanzania | 2013 | Canis lupus familiaris |
| KY210272 | Tanzania | 2013 | Canis lupus familiaris |
| KY210273 | Tanzania | 2013 | Canis lupus familiaris |
| KY210274 | Tanzania | 2013 | Bos taurus |
| KY210277 | Tanzania | 2011 | Bos taurus |
| KY210278 | Tanzania | 2011 | Canis lupus familiaris |
| KY210281 | Tanzania | 2012 | Canis lupus familiaris |
| KY210282 | Tanzania | 2012 | Canis lupus familiaris |
| KY210283 | Tanzania | 2012 | Bos taurus |
| KY210284 | Tanzania | 2012 | Canis |
| KY210285 | Tanzania | 2012 | Capra hircus |
| KY210286 | Tanzania | 2011 | Canis lupus familiaris |
| KY210288 | Tanzania | 2012 | Canis lupus familiaris |
| KY210289 | Tanzania | 2012 | Canis lupus familiaris |
| KY210290 | Tanzania | 2012 | Canis lupus familiaris |
| KY210291 | Tanzania | 2012 | Canis |
| KY210292 | Tanzania | 2012 | Canis lupus familiaris |
| KY210293 | Tanzania | 2012 | Canis lupus familiaris |
| KY210295 | Tanzania | 2012 | Canis lupus familiaris |
| KY210296 | Tanzania | 2012 | Canis lupus familiaris |
| KY210297 | Tanzania | 2012 | Ovis aries |
| KY210298 | Tanzania | 2012 | Capra hircus |
| KY210299 | Tanzania | 2012 | Canis lupus familiaris |
| KY210300 | Tanzania | 2012 | Canis lupus familiaris |
| KY210301 | Tanzania | 2012 | Bos taurus |
| KY210302 | Tanzania | 2012 | Capra hircus |
| KY210303 | Tanzania | 2012 | Canis lupus familiaris |
| KY210305 | Tanzania | 2012 | Hyaenidae |
| KY210307 | Tanzania | 2012 | Canis lupus familiaris |
| KY210311 | Tanzania | 2012 | Canis lupus familiaris |
| KY451767 | China | 2015 | Canis lupus familiaris |
| KY765901 | Tajikistan | 2012 | Canis lupus familiaris |
| KY775603 | India | 2014 | Boselaphus tragocamelus |
| KY775604 | India | 2014 | Herpestidae |
| KY780299 | China | 2017 | Homo sapiens |
| KY860583 | Turkey | 1999 | Canis lupus familiaris |
| KY860584 | Turkey | 1999 | Canidae |
| KY860585 | Turkey | 2001 | Canidae |
| KY860586 | Turkey | 2001 | Canidae |
| KY860587 | Turkey | 2001 | Canidae |
| KY860588 | Turkey | 2004 | Canidae |
| KY860589 | Turkey | 2006 | Canidae |
| KY860590 | Turkey | 2007 | Canidae |
| KY860591 | Turkey | 2008 | Canidae |
| KY860592 | Turkey | 2009 | Canidae |
| KY860593 | Turkey | 2010 | Canidae |
| KY860594 | Turkey | 2012 | Canidae |
| KY860595 | Turkey | 2012 | Canidae |
| KY860596 | Turkey | 2014 | Canidae |
| KY860597 | Turkey | 2014 | Canis lupus familiaris |
| KY860598 | Turkey | 2014 | Canidae |
| KY860599 | Turkey | 2014 | Canis lupus familiaris |
| KY860600 | Turkey | 2014 | Canidae |
| KY860601 | Turkey | 2014 | Canidae |
| KY860602 | Turkey | 2014 | Canis lupus familiaris |
| KY860603 | Turkey | 2015 | Canidae |
| KY860604 | Turkey | 2000 | Canidae |
| KY860605 | Turkey | 2001 | Canidae |
| KY860606 | Turkey | 2001 | Canidae |
| KY860607 | Turkey | 2001 | Canis lupus familiaris |
| KY860608 | Turkey | 2001 | Canis lupus familiaris |
| KY860609 | Turkey | 2001 | Canis lupus familiaris |
| KY860610 | Turkey | 2001 | Canis lupus familiaris |
| KY860611 | Turkey | 2001 | Canis lupus familiaris |
| KY860612 | Turkey | 1989 | Canis lupus familiaris |
| KY860613 | Russia | 1989 | Nyctereutes procyonoides |
| KY912036 | China | 2014 | Bos grunniens |
| KY952219 | China | 2014 | Canis |
| KY952220 | China | 2016 | Canis lupus familiaris |
| KY964322 | China | 2016 | Canis lupus familiaris |
| KY964323 | China | 2016 | Homo sapiens |
| KY982922 | China | 2014 | Canis lupus familiaris |
| KY982923 | China | 2012 | Homo sapiens |
| KY997452 | China | 2014 | Canis lupus familiaris |
| LC571945 | Japan | 2006 | Homo sapiens |
| LC619707 | Japan | 2020 | Homo sapiens |
| LN879480 | Azerbaijan | 2002 | Canis lupus familiaris |
| LN879481 | Germany | 1998 | Vulpes vulpes |
| LT598537 | Canada | 1977 | Vulpes lagopus |
| LT598538 | Greenland | 2013 | Vulpes lagopus |
| LT598539 | Greenland | 2013 | Vulpes lagopus |
| LT598540 | Greenland | 2014 | Vulpes lagopus |
| LT598541 | Greenland | 2013 | Vulpes lagopus |
| LT598542 | Greenland | 2014 | Vulpes lagopus |
| LT598543 | Greenland | 2014 | Vulpes lagopus |
| LT909526 | Iran | 1991 | Vulpes vulpes |
| LT909527 | Indonesia | 1988 | Canis lupus familiaris |
| LT909528 | Tanzania | 2009 | Vulpes vulpes |
| LT909529 | Algeria | 1984 | Canis lupus familiaris |
| LT909530 | Chile | 1979 | Homo sapiens |
| LT909531 | Sudan | 1993 | Canis lupus familiaris |
| LT909532 | Poland | 1994 | Vulpes vulpes |
| LT909533 | Saudi Arabia | 1990 | Vulpes vulpes |
| LT909534 | Kenya | 1997 | Vulpes vulpes |
| LT909535 | Algeria | 1989 | Canis lupus familiaris |
| LT909536 | Kenya | 2001 | Canis lupus familiaris |
| LT909537 | Estonia | 2000 | Vulpes vulpes |
| LT909538 | Finland | 1990 | Vulpes vulpes |
| LT909539 | India | 2002 | Canis lupus familiaris |
| LT909540 | Saudi Arabia | 1987 | Vulpes vulpes |
| LT909541 | Pakistan | 1984 | Canis lupus familiaris |
| LT909542 | Algeria | 1984 | Canis lupus familiaris |
| LT909543 | Chile | 1973 | Homo sapiens |
| LT909544 | Mexico | 2002 | Canis lupus familiaris |
| LT909545 | Afghanistan | 2006 | Canis lupus familiaris |
| LT909546 | Nigeria | 1988 | Felis catus |
| LT909547 | Germany | 1990 | Vulpes vulpes |
| LT909548 | Nigeria | 1989 | Canis lupus familiaris |
| LT909550 | Norway | 2000 | Vulpes vulpes |
| LT909551 | Ethiopia | 1992 | Canis lupus familiaris |
| LT993233 | Germany | 2001 | Vulpes vulpes |
| LT993235 | Slovenia | 2012 | Vulpes vulpes |
| LT993236 | Slovenia | 2014 | Martes foina |
| LT993237 | Latvia | 2013 | Meles meles |
| LT993238 | Latvia | 2013 | Meles meles |
| LT993239 | Slovenia | 2012 | Vulpes vulpes |
| LT993240 | Canada | 1991 | Mephitis mephitis |
| LT993241 | Canada | 1994 | Bos taurus |
| LT993242 | Canada | 1996 | Vulpes vulpes |
| LT993243 | Austria | 2004 | Vulpes vulpes |
| LT993244 | Germany | 2002 | Vulpes vulpes |
| LT993245 | Romania | 2015 | Bos taurus |
| LT993246 | Austria | 2004 | Vulpes vulpes |
| MF143192 | USA | 2013 | Procyon lotor |
| MF143193 | USA | 2013 | Procyon lotor |
| MF143195 | USA | 2013 | Procyon lotor |
| MF143196 | USA | 2013 | Procyon lotor |
| MF143197 | USA | 2013 | Mephitidae |
| MF143198 | USA | 2013 | Mephitidae |
| MF143199 | USA | 2013 | Procyon lotor |
| MF143200 | USA | 2013 | Procyon lotor |
| MF143201 | USA | 2013 | Procyon lotor |
| MF143202 | USA | 2013 | Mephitidae |
| MF143206 | USA | 2013 | Procyon lotor |
| MF143207 | USA | 2013 | Urocyon cinereoargenteus |
| MF143208 | USA | 2013 | Procyon lotor |
| MF143209 | USA | 2013 | Mephitidae |
| MF143211 | USA | 2013 | Mephitidae |
| MF143212 | USA | 2013 | Mephitidae |
| MF143213 | USA | 2014 | Procyon lotor |
| MF143214 | USA | 2014 | Mephitidae |
| MF143215 | USA | 2014 | Procyon lotor |
| MF143217 | USA | 2014 | Procyon lotor |
| MF143218 | USA | 2014 | Procyon lotor |
| MF143219 | USA | 2014 | Procyon lotor |
| MF143220 | USA | 2014 | Urocyon cinereoargenteus |
| MF143221 | USA | 2014 | Procyon lotor |
| MF143222 | USA | 2014 | Procyon lotor |
| MF143223 | Canada | 2000 | Mephitidae |
| MF143224 | Canada | 2000 | Mephitidae |
| MF143225 | Canada | 2000 | Procyon lotor |
| MF143226 | Canada | 2000 | Mephitidae |
| MF143227 | Canada | 2000 | Procyon lotor |
| MF143229 | Canada | 2001 | Mephitidae |
| MF143230 | Canada | 2001 | Procyon lotor |
| MF143232 | Canada | 2001 | Procyon lotor |
| MF143233 | Canada | 2001 | Procyon lotor |
| MF143234 | Canada | 2002 | Procyon lotor |
| MF143235 | Canada | 2014 | Procyon lotor |
| MF143237 | Canada | 2015 | Procyon lotor |
| MF143238 | Canada | 2015 | Procyon lotor |
| MF143239 | Canada | 2015 | Procyon lotor |
| MF143241 | Canada | 2015 | Procyon lotor |
| MF143242 | Canada | 2015 | Procyon lotor |
| MF143243 | Canada | 2015 | Procyon lotor |
| MF143244 | Canada | 2015 | Procyon lotor |
| MF143245 | Canada | 2015 | Procyon lotor |
| MF143246 | Canada | 2015 | Procyon lotor |
| MF143247 | Canada | 2015 | Procyon lotor |
| MF143248 | Canada | 2015 | Procyon lotor |
| MF143249 | Canada | 2015 | Procyon lotor |
| MF143250 | Canada | 2015 | Procyon lotor |
| MF143251 | Canada | 2015 | Mephitidae |
| MF143252 | Canada | 2015 | Procyon lotor |
| MF143253 | Canada | 2015 | Procyon lotor |
| MF143254 | Canada | 2015 | Procyon lotor |
| MF143256 | Canada | 2007 | Procyon lotor |
| MF143257 | USA | 2004 | Procyon lotor |
| MF143258 | USA | 2004 | Procyon lotor |
| MF143259 | Canada | 2007 | Procyon lotor |
| MF143260 | Canada | 2007 | Procyon lotor |
| MF143261 | Canada | 2007 | Procyon lotor |
| MF143262 | Canada | 2007 | Procyon lotor |
| MF143263 | Canada | 2007 | Procyon lotor |
| MF143265 | Canada | 2007 | Procyon lotor |
| MF143266 | Canada | 2007 | Procyon lotor |
| MF143267 | Canada | 2007 | Procyon lotor |
| MF143268 | Canada | 2007 | Procyon lotor |
| MF143269 | Canada | 2007 | Procyon lotor |
| MF143270 | Canada | 2007 | Procyon lotor |
| MF143271 | Canada | 2007 | Mephitidae |
| MF143272 | Canada | 2008 | Procyon lotor |
| MF143273 | Canada | 2008 | Procyon lotor |
| MF143275 | Canada | 2008 | Mephitidae |
| MF143276 | Canada | 2009 | Mephitidae |
| MF143278 | USA | 2006 | Bos taurus |
| MF143279 | USA | 2006 | Procyon lotor |
| MF143280 | USA | 2006 | Mephitidae |
| MF143281 | USA | 2006 | Mephitidae |
| MF143283 | USA | 2007 | Procyon lotor |
| MF143284 | USA | 2007 | Procyon lotor |
| MF143285 | USA | 2007 | Mephitidae |
| MF143286 | USA | 2007 | Procyon lotor |
| MF143287 | USA | 2007 | Procyon lotor |
| MF143288 | USA | 2008 | Mephitidae |
| MF143289 | USA | 2009 | Procyon lotor |
| MF143290 | USA | 1994 | Procyon lotor |
| MF143291 | USA | 1995 | Procyon lotor |
| MF143292 | USA | 2003 | Procyon lotor |
| MF143293 | USA | 2003 | Procyon lotor |
| MF143294 | USA | 2003 | Mephitidae |
| MF143295 | USA | 2003 | Procyon lotor |
| MF143297 | USA | 2004 | Procyon lotor |
| MF143298 | USA | 2004 | Procyon lotor |
| MF143300 | USA | 2004 | Procyon lotor |
| MF143301 | USA | 2004 | Procyon lotor |
| MF143302 | USA | 2004 | Procyon lotor |
| MF143303 | USA | 2004 | Procyon lotor |
| MF143304 | USA | 2004 | Procyon lotor |
| MF143305 | USA | 2004 | Mephitidae |
| MF143306 | USA | 2004 | Mephitidae |
| MF143307 | USA | 2004 | Mephitidae |
| MF143308 | USA | 2010 | Mephitidae |
| MF143309 | USA | 2010 | Procyon lotor |
| MF143310 | USA | 2010 | Procyon lotor |
| MF143311 | USA | 2010 | Mephitidae |
| MF143312 | USA | 2010 | Procyon lotor |
| MF143313 | USA | 2010 | Procyon lotor |
| MF143314 | USA | 2010 | Procyon lotor |
| MF143315 | USA | 2010 | Procyon lotor |
| MF143317 | USA | 2010 | Mephitidae |
| MF143318 | USA | 2014 | Procyon lotor |
| MF143320 | USA | 2010 | Procyon lotor |
| MF143321 | USA | 2010 | Mephitidae |
| MF143322 | USA | 2010 | Procyon lotor |
| MF143323 | USA | 2010 | Mephitidae |
| MF143324 | USA | 2011 | Procyon lotor |
| MF143325 | USA | 2011 | Procyon lotor |
| MF143326 | USA | 2011 | Procyon lotor |
| MF143327 | USA | 2011 | Felis catus |
| MF143328 | USA | 2011 | Procyon lotor |
| MF143329 | USA | 2011 | Procyon lotor |
| MF143331 | USA | 2011 | Mephitidae |
| MF143332 | USA | 2011 | Mephitidae |
| MF143333 | USA | 2011 | Felis catus |
| MF143334 | USA | 2011 | Mephitidae |
| MF143335 | USA | 2011 | Urocyon cinereoargenteus |
| MF143336 | USA | 2003 | Procyon lotor |
| MF143337 | USA | 2007 | Mephitidae |
| MF143338 | USA | 2009 | Bos taurus |
| MF143339 | USA | 2010 | Mephitidae |
| MF143340 | USA | 2011 | Mephitidae |
| MF143341 | Canada | 2006 | Procyon lotor |
| MF143342 | Canada | 2006 | Procyon lotor |
| MF143343 | Canada | 2006 | Procyon lotor |
| MF143344 | Canada | 2007 | Procyon lotor |
| MF143345 | Canada | 2007 | Procyon lotor |
| MF143347 | Canada | 2007 | Procyon lotor |
| MF143348 | Canada | 2007 | Procyon lotor |
| MF143349 | Canada | 2007 | Procyon lotor |
| MF143350 | Canada | 2007 | Procyon lotor |
| MF143351 | Canada | 2007 | Procyon lotor |
| MF143352 | Canada | 2007 | Procyon lotor |
| MF143353 | Canada | 2007 | Procyon lotor |
| MF143354 | Canada | 2007 | Procyon lotor |
| MF143355 | Canada | 2007 | Procyon lotor |
| MF143356 | Canada | 2007 | Procyon lotor |
| MF143357 | Canada | 2007 | Procyon lotor |
| MF143358 | Canada | 2007 | Mephitidae |
| MF143359 | Canada | 2007 | Vulpes vulpes |
| MF143360 | Canada | 2007 | Procyon lotor |
| MF143361 | Canada | 2008 | Procyon lotor |
| MF143362 | Canada | 2008 | Procyon lotor |
| MF143363 | Canada | 2008 | Procyon lotor |
| MF143364 | Canada | 2008 | Procyon lotor |
| MF143365 | Canada | 2008 | Procyon lotor |
| MF143366 | Canada | 2008 | Procyon lotor |
| MF143367 | Canada | 2008 | Mephitidae |
| MF143368 | Canada | 2008 | Procyon lotor |
| MF143369 | Canada | 2008 | Procyon lotor |
| MF143370 | Canada | 2008 | Procyon lotor |
| MF143371 | Canada | 2008 | Procyon lotor |
| MF143372 | Canada | 2009 | Mephitidae |
| MF143373 | USA | 2005 | Procyon lotor |
| MF143375 | USA | 2006 | Procyon lotor |
| MF143376 | USA | 2006 | Mephitidae |
| MF143377 | USA | 2006 | Mephitidae |
| MF143378 | USA | 2007 | Mephitidae |
| MF143379 | USA | 2007 | Procyon lotor |
| MF143380 | USA | 2007 | Procyon lotor |
| MF143381 | USA | 2007 | Procyon lotor |
| MF143382 | USA | 2007 | Mephitidae |
| MF143383 | USA | 2007 | Mephitidae |
| MF143384 | USA | 2007 | Mephitidae |
| MF143385 | USA | 2007 | Procyon lotor |
| MF143386 | USA | 2007 | Procyon lotor |
| MF143387 | USA | 2007 | Mephitidae |
| MF143388 | USA | 2007 | Mephitidae |
| MF143389 | USA | 2007 | Mephitidae |
| MF143390 | USA | 2007 | Mephitidae |
| MF143391 | USA | 2008 | Bos taurus |
| MF143392 | USA | 2009 | Mephitidae |
| MF143395 | USA | 2009 | Mephitidae |
| MF143396 | USA | 2009 | Mephitidae |
| MF143397 | USA | 2009 | Procyon lotor |
| MF143398 | USA | 2009 | Procyon lotor |
| MF143399 | USA | 2009 | Procyon lotor |
| MF143400 | USA | 2009 | Mephitidae |
| MF143402 | USA | 2009 | Procyon lotor |
| MF143403 | USA | 2009 | Bos taurus |
| MF143404 | USA | 2009 | Procyon lotor |
| MF143405 | USA | 2009 | Procyon lotor |
| MF143406 | USA | 2009 | Bos taurus |
| MF143407 | USA | 2010 | Mephitidae |
| MF143408 | USA | 2010 | Procyon lotor |
| MF143409 | USA | 2010 | Procyon lotor |
| MF143410 | USA | 2010 | Mephitidae |
| MF143411 | USA | 2010 | Mephitidae |
| MF143412 | USA | 2010 | Mephitidae |
| MF143413 | USA | 2010 | Mephitidae |
| MF143415 | USA | 2011 | Procyon lotor |
| MF197741 | Poland | 2008 | Canidae |
| MF197742 | Poland | 2014 | Canidae |
| MF197743 | Poland | 2010 | Canidae |
| MF197744 | Poland | 2014 | Eptesicus serotinus |
| MF197745 | Poland | 1998 | Eptesicus serotinus |
| MG458304 | USA | 1975 | Chiroptera |
| MG458307 | South Africa | 2000 | Felis catus |
| MG458308 | South Africa | 2000 | Herpestidae |
| MG458309 | Serbia | 1978 | Canis lupus familiaris |
| MG458310 | Serbia | 1986 | Bos taurus |
| MG458311 | Serbia | 1998 | Canidae |
| MG458312 | Serbia | 1997 | Canidae |
| MG458313 | Russia | 1996 | Vulpes lagopus |
| MG458315 | Egypt | 1998 | Canis lupus familiaris |
| MG458316 | Egypt | 1998 | Canis lupus familiaris |
| MG458317 | Egypt | 1999 | Canis lupus familiaris |
| MG458318 | South Africa | 2008 | Homo sapiens |
| MG458319 | Grenada | 2012 | Herpestidae |
| MG458320 | Nepal | 2012 | Homo sapiens |
| MG562518 | USA | 2003 | Procyon lotor |
| MG562519 | USA | 2003 | Procyon lotor |
| MG562520 | USA | 2003 | Procyon lotor |
| MG562521 | USA | 2004 | Equus caballus |
| MG562522 | USA | 2003 | Procyon lotor |
| MG562523 | USA | 1990 | Procyon lotor |
| MG562525 | USA | 1992 | Procyon lotor |
| MG562526 | USA | 1995 | Procyon lotor |
| MG562527 | USA | 2003 | Procyon lotor |
| MG562530 | USA | 2003 | Procyon lotor |
| MG562531 | USA | 2003 | Procyon lotor |
| MG562532 | USA | 2003 | Mephitidae |
| MG562533 | USA | 2003 | Procyon lotor |
| MG562535 | USA | 2004 | Procyon lotor |
| MG562536 | USA | 2004 | Procyon lotor |
| MG562537 | USA | 2004 | Procyon lotor |
| MG562538 | USA | 2004 | Procyon lotor |
| MG562539 | USA | 2004 | Procyon lotor |
| MG562540 | USA | 2004 | Mephitidae |
| MG562542 | USA | 2004 | Procyon lotor |
| MG562543 | USA | 2010 | Procyon lotor |
| MG562545 | USA | 2010 | Mephitidae |
| MG562546 | USA | 2010 | Procyon lotor |
| MG562547 | USA | 2011 | Mephitidae |
| MG562548 | USA | 2011 | Procyon lotor |
| MG562549 | USA | 2011 | Procyon lotor |
| MG562550 | USA | 2011 | Procyon lotor |
| MG562551 | USA | 2011 | Urocyon cinereoargenteus |
| MG562553 | USA | 2011 | Procyon lotor |
| MG562554 | USA | 2011 | Mephitidae |
| MG562555 | USA | 2005 | Procyon lotor |
| MG562556 | USA | 2005 | Procyon lotor |
| MG562557 | USA | 2005 | Procyon lotor |
| MG562559 | USA | 2005 | Procyon lotor |
| MG562560 | USA | 2005 | Procyon lotor |
| MG562561 | USA | 2005 | Procyon lotor |
| MG562562 | USA | 2005 | Procyon lotor |
| MG562563 | USA | 2005 | Procyon lotor |
| MG562564 | USA | 2006 | Procyon lotor |
| MG562565 | USA | 2006 | Procyon lotor |
| MG562566 | USA | 2006 | Procyon lotor |
| MG562567 | USA | 2006 | Mephitidae |
| MG562568 | USA | 2006 | Procyon lotor |
| MG562569 | USA | 2006 | Procyon lotor |
| MG562571 | USA | 2006 | Bos taurus |
| MG562572 | USA | 2006 | Procyon lotor |
| MG562573 | USA | 2006 | Procyon lotor |
| MG562574 | USA | 2006 | Marmota monax |
| MG562577 | USA | 2007 | Mephitidae |
| MG562578 | USA | 2007 | Procyon lotor |
| MG562579 | USA | 2007 | Mephitidae |
| MG562580 | USA | 2008 | Procyon lotor |
| MG562581 | USA | 2008 | Procyon lotor |
| MG562582 | USA | 2008 | Mephitidae |
| MG562583 | USA | 2008 | Procyon lotor |
| MG562584 | USA | 2008 | Mephitidae |
| MG562585 | USA | 2008 | Mephitidae |
| MG562586 | USA | 2008 | Mephitidae |
| MG562587 | USA | 2008 | Mephitidae |
| MG562588 | USA | 2008 | Mephitidae |
| MG562589 | USA | 2008 | Procyon lotor |
| MG562590 | USA | 2008 | Procyon lotor |
| MG562591 | USA | 2008 | Procyon lotor |
| MG562592 | USA | 2008 | Procyon lotor |
| MG562593 | USA | 2009 | Mephitidae |
| MG562594 | USA | 2009 | Equus caballus |
| MG562595 | USA | 2009 | Procyon lotor |
| MG562596 | USA | 2009 | Urocyon cinereoargenteus |
| MG562597 | USA | 2009 | Procyon lotor |
| MG562598 | USA | 2009 | Procyon lotor |
| MG562599 | USA | 2009 | Procyon lotor |
| MG562600 | USA | 2009 | Procyon lotor |
| MG562601 | USA | 2009 | Procyon lotor |
| MG562602 | USA | 2009 | Procyon lotor |
| MG562603 | USA | 2009 | Bos taurus |
| MG562604 | USA | 2009 | Procyon lotor |
| MG562605 | USA | 2009 | Mephitidae |
| MG562606 | USA | 2009 | Procyon lotor |
| MG562607 | USA | 2010 | Urocyon cinereoargenteus |
| MG562608 | USA | 2010 | Mephitidae |
| MG562609 | USA | 2010 | Urocyon cinereoargenteus |
| MG562610 | USA | 2011 | Mephitidae |
| MH267792 | China | 2017 | Ovis aries |
| MH514968 | Senegal | 2001 | Homo sapiens |
| MH514969 | Senegal | 2005 | Homo sapiens |
| MH514970 | Senegal | 2011 | Canis lupus familiaris |
| MH514971 | Senegal | 2010 | Canis lupus familiaris |
| MH514972 | Senegal | 2008 | Canis lupus familiaris |
| MH514973 | Senegal | 2011 | Mellivora capensis |
| MH514974 | Senegal | 2011 | Canis lupus familiaris |
| MH514975 | Senegal | 2013 | Canis lupus familiaris |
| MH514976 | Senegal | 2010 | Canis lupus familiaris |
| MH514977 | Senegal | 2013 | Canis lupus familiaris |
| MH514978 | Senegal | 2014 | Canis lupus familiaris |
| MH514979 | Senegal | 2014 | Canis lupus familiaris |
| MH514980 | Senegal | 2014 | Canis lupus familiaris |
| MH514981 | Senegal | 2013 | Canis lupus familiaris |
| MH514982 | Senegal | 2007 | Homo sapiens |
| MH514983 | Senegal | 2011 | Canis lupus familiaris |
| MH514984 | Senegal | 2011 | Homo sapiens |
| MH514985 | Senegal | 2015 | Canis lupus familiaris |
| MH671332 | China | 2017 | Homo sapiens |
| MK111075 | Hungary | 2015 | Canidae |
| MK540658 | USA | 1990 | Procyon lotor |
| MK540659 | USA | 1990 | Procyon lotor |
| MK540660 | USA | 1990 | Procyon lotor |
| MK540661 | USA | 1990 | Procyon lotor |
| MK540662 | USA | 1990 | Procyon lotor |
| MK540664 | USA | 1990 | Procyon lotor |
| MK540665 | USA | 1990 | Procyon lotor |
| MK540666 | USA | 1990 | Procyon lotor |
| MK540667 | USA | 1990 | Procyon lotor |
| MK540668 | USA | 1990 | Mephitidae |
| MK540669 | USA | 1990 | Bos taurus |
| MK540670 | USA | 1990 | Procyon lotor |
| MK540671 | USA | 1990 | Procyon lotor |
| MK540672 | USA | 1990 | Procyon lotor |
| MK540673 | USA | 1990 | Procyon lotor |
| MK540674 | USA | 1990 | Procyon lotor |
| MK540675 | USA | 1990 | Procyon lotor |
| MK540676 | USA | 1990 | Procyon lotor |
| MK540677 | USA | 1990 | Procyon lotor |
| MK540678 | USA | 1991 | Procyon lotor |
| MK540679 | USA | 1991 | Procyon lotor |
| MK540680 | USA | 1991 | Procyon lotor |
| MK540681 | USA | 1991 | Procyon lotor |
| MK540682 | USA | 1992 | Procyon lotor |
| MK540683 | USA | 1992 | Procyon lotor |
| MK540684 | USA | 1992 | Procyon lotor |
| MK540685 | USA | 1992 | Procyon lotor |
| MK540687 | USA | 1992 | Procyon lotor |
| MK540688 | USA | 1993 | Procyon lotor |
| MK540689 | USA | 1993 | Procyon lotor |
| MK540690 | USA | 1995 | Procyon lotor |
| MK540691 | USA | 2003 | Procyon lotor |
| MK540692 | USA | 2003 | Procyon lotor |
| MK540693 | USA | 2003 | Procyon lotor |
| MK540694 | USA | 2003 | Procyon lotor |
| MK540695 | USA | 2003 | Mephitidae |
| MK540696 | USA | 2004 | Mephitidae |
| MK540697 | USA | 2004 | Mephitidae |
| MK540698 | USA | 2004 | Procyon lotor |
| MK540699 | USA | 2004 | Procyon lotor |
| MK540700 | USA | 2004 | Procyon lotor |
| MK540701 | USA | 2004 | Procyon lotor |
| MK540702 | USA | 2004 | Procyon lotor |
| MK540703 | USA | 2004 | Procyon lotor |
| MK540705 | USA | 2004 | Procyon lotor |
| MK540706 | USA | 2004 | Procyon lotor |
| MK540708 | USA | 2004 | Procyon lotor |
| MK540709 | USA | 2004 | Procyon lotor |
| MK540711 | USA | 2004 | Procyon lotor |
| MK540712 | USA | 2004 | Mephitidae |
| MK540714 | USA | 2004 | Procyon lotor |
| MK540715 | USA | 2004 | Procyon lotor |
| MK540716 | USA | 2004 | Mephitidae |
| MK540717 | USA | 2004 | Procyon lotor |
| MK540718 | USA | 2004 | Procyon lotor |
| MK540719 | USA | 2004 | Procyon lotor |
| MK540720 | USA | 2004 | Mephitidae |
| MK540721 | USA | 2004 | Procyon lotor |
| MK540723 | USA | 2010 | Procyon lotor |
| MK540724 | USA | 2010 | Procyon lotor |
| MK540725 | USA | 2010 | Procyon lotor |
| MK540727 | USA | 2010 | Procyon lotor |
| MK540728 | USA | 2010 | Procyon lotor |
| MK540730 | USA | 2010 | Procyon lotor |
| MK540731 | USA | 2010 | Procyon lotor |
| MK540732 | USA | 2010 | Procyon lotor |
| MK540733 | USA | 2010 | Procyon lotor |
| MK540734 | USA | 2010 | Procyon lotor |
| MK540735 | USA | 2010 | Procyon lotor |
| MK540736 | USA | 2010 | Procyon lotor |
| MK540737 | USA | 2010 | Mephitidae |
| MK540738 | USA | 2010 | Mephitidae |
| MK540739 | USA | 2010 | Procyon lotor |
| MK540740 | USA | 2010 | Mephitidae |
| MK540741 | USA | 2010 | Procyon lotor |
| MK540742 | USA | 2011 | Mephitidae |
| MK540743 | USA | 2011 | Procyon lotor |
| MK540744 | USA | 2011 | Procyon lotor |
| MK540745 | USA | 2011 | Procyon lotor |
| MK540746 | USA | 2011 | Procyon lotor |
| MK540747 | USA | 2011 | Urocyon cinereoargenteus |
| MK540748 | USA | 2011 | Procyon lotor |
| MK540749 | USA | 2011 | Procyon lotor |
| MK540750 | USA | 2011 | Procyon lotor |
| MK540751 | USA | 2011 | Procyon lotor |
| MK540753 | USA | 2011 | Procyon lotor |
| MK540754 | USA | 2011 | Procyon lotor |
| MK540756 | USA | 2011 | Procyon lotor |
| MK540757 | USA | 2011 | Urocyon cinereoargenteus |
| MK540758 | USA | 2011 | Procyon lotor |
| MK540759 | USA | 2011 | Felis catus |
| MK540760 | USA | 2011 | Felis catus |
| MK540762 | USA | 2011 | Felis catus |
| MK540763 | USA | 2011 | Procyon lotor |
| MK540764 | USA | 2011 | Vulpes vulpes |
| MK540766 | USA | 2011 | Urocyon cinereoargenteus |
| MK540767 | USA | 2011 | Procyon lotor |
| MK540768 | USA | 2011 | Procyon lotor |
| MK540769 | USA | 2011 | Felis catus |
| MK540770 | USA | 2011 | Procyon lotor |
| MK540771 | USA | 2011 | Procyon lotor |
| MK540772 | USA | 2011 | Felis catus |
| MK540773 | USA | 2011 | Procyon lotor |
| MK540774 | USA | 2011 | Felis catus |
| MK540775 | USA | 2011 | Procyon lotor |
| MK540776 | USA | 2011 | Urocyon cinereoargenteus |
| MK540777 | USA | 2011 | Felis catus |
| MK540778 | USA | 2011 | Procyon lotor |
| MK540779 | USA | 2011 | Procyon lotor |
| MK540780 | USA | 2011 | Mephitidae |
| MK540781 | USA | 2011 | Marmota monax |
| MK540784 | USA | 2011 | Procyon lotor |
| MK540785 | USA | 2011 | Procyon lotor |
| MK540786 | USA | 2011 | Urocyon cinereoargenteus |
| MK540787 | USA | 2011 | Mephitidae |
| MK540789 | USA | 2011 | Urocyon cinereoargenteus |
| MK540790 | USA | 2011 | Bos taurus |
| MK540791 | USA | 2011 | Felis catus |
| MK540794 | USA | 2015 | Procyon lotor |
| MK540795 | USA | 2015 | Procyon lotor |
| MK540796 | USA | 2015 | Procyon lotor |
| MK540797 | USA | 2018 | Procyon lotor |
| MK540798 | USA | 2018 | Procyon lotor |
| MK540799 | USA | 2018 | Procyon lotor |
| MK540801 | Canada | 2015 | Procyon lotor |
| MK540802 | Canada | 2015 | Procyon lotor |
| MK540803 | Canada | 2015 | Procyon lotor |
| MK540804 | Canada | 2015 | Procyon lotor |
| MK540805 | Canada | 2016 | Procyon lotor |
| MK540806 | Canada | 2016 | Procyon lotor |
| MK540807 | Canada | 2016 | Procyon lotor |
| MK540808 | Canada | 2016 | Procyon lotor |
| MK540809 | Canada | 2016 | Procyon lotor |
| MK540810 | Canada | 2016 | Procyon lotor |
| MK540811 | Canada | 2016 | Procyon lotor |
| MK540812 | Canada | 2016 | Mephitidae |
| MK540813 | Canada | 2016 | Procyon lotor |
| MK540814 | Canada | 2016 | Mephitidae |
| MK540815 | Canada | 2016 | Procyon lotor |
| MK540816 | Canada | 2016 | Mephitidae |
| MK540817 | Canada | 2016 | Mephitidae |
| MK540818 | Canada | 2016 | Procyon lotor |
| MK540819 | Canada | 2016 | Mephitidae |
| MK540820 | Canada | 2016 | Procyon lotor |
| MK540821 | Canada | 2016 | Mephitidae |
| MK540822 | Canada | 2016 | Mephitidae |
| MK540823 | Canada | 2016 | Procyon lotor |
| MK540826 | Canada | 2016 | Procyon lotor |
| MK540827 | Canada | 2016 | Procyon lotor |
| MK540828 | Canada | 2016 | Procyon lotor |
| MK540829 | Canada | 2016 | Procyon lotor |
| MK540830 | Canada | 2016 | Procyon lotor |
| MK540831 | Canada | 2016 | Procyon lotor |
| MK540832 | Canada | 2016 | Procyon lotor |
| MK540833 | Canada | 2016 | Mephitidae |
| MK540834 | Canada | 2016 | Procyon lotor |
| MK540836 | Canada | 2016 | Mephitidae |
| MK540837 | Canada | 2016 | Procyon lotor |
| MK540838 | Canada | 2016 | Procyon lotor |
| MK540839 | Canada | 2016 | Procyon lotor |
| MK540840 | Canada | 2016 | Procyon lotor |
| MK540841 | Canada | 2016 | Mephitidae |
| MK540842 | Canada | 2016 | Procyon lotor |
| MK540843 | Canada | 2016 | Vulpes vulpes |
| MK540845 | Canada | 2016 | Mephitidae |
| MK540846 | Canada | 2016 | Procyon lotor |
| MK540847 | Canada | 2016 | Procyon lotor |
| MK540848 | Canada | 2016 | Procyon lotor |
| MK540849 | Canada | 2016 | Mephitidae |
| MK540850 | Canada | 2016 | Procyon lotor |
| MK540852 | Canada | 2016 | Procyon lotor |
| MK540853 | Canada | 2016 | Procyon lotor |
| MK540854 | Canada | 2016 | Procyon lotor |
| MK540855 | Canada | 2016 | Procyon lotor |
| MK540856 | Canada | 2016 | Procyon lotor |
| MK540857 | Canada | 2016 | Procyon lotor |
| MK540858 | Canada | 2016 | Mephitidae |
| MK540859 | Canada | 2016 | Procyon lotor |
| MK540861 | Canada | 2016 | Mephitidae |
| MK540863 | Canada | 2016 | Mephitidae |
| MK540864 | Canada | 2016 | Procyon lotor |
| MK540865 | Canada | 2016 | Procyon lotor |
| MK540866 | Canada | 2016 | Mephitidae |
| MK540867 | Canada | 2016 | Mephitidae |
| MK540868 | Canada | 2016 | Procyon lotor |
| MK540869 | Canada | 2016 | Procyon lotor |
| MK540870 | Canada | 2016 | Mephitidae |
| MK540871 | Canada | 2016 | Mephitidae |
| MK540874 | Canada | 2017 | Procyon lotor |
| MK540875 | Canada | 2017 | Procyon lotor |
| MK540876 | Canada | 2017 | Procyon lotor |
| MK540877 | Canada | 2017 | Mephitidae |
| MK540880 | Canada | 2017 | Mephitidae |
| MK540881 | Canada | 2017 | Mephitidae |
| MK540882 | Canada | 2017 | Procyon lotor |
| MK540883 | USA | 1989 | Procyon lotor |
| MK540884 | USA | 1989 | Procyon lotor |
| MK540885 | USA | 1989 | Procyon lotor |
| MK540886 | USA | 1989 | Procyon lotor |
| MK540895 | Canada | 1991 | Vulpes vulpes |
| MK540896 | Canada | 1991 | Mephitidae |
| MK540897 | Canada | 1991 | Mephitidae |
| MK540898 | Canada | 1991 | Vulpes vulpes |
| MK540899 | Canada | 1991 | Vulpes vulpes |
| MK540902 | Canada | 1993 | Mephitidae |
| MK540903 | Canada | 1993 | Vulpes vulpes |
| MK540904 | Canada | 1993 | Vulpes vulpes |
| MK540905 | Canada | 1993 | Vulpes vulpes |
| MK540906 | Canada | 1995 | Vulpes vulpes |
| MK540907 | Canada | 1995 | Mephitidae |
| MK540908 | Canada | 1995 | Vulpes vulpes |
| MK540909 | Canada | 1995 | Vulpes vulpes |
| MK540911 | Canada | 1995 | Vulpes vulpes |
| MK540912 | Canada | 1995 | Mephitidae |
| MK540913 | Canada | 1996 | Mephitidae |
| MK540914 | Canada | 1996 | Vulpes vulpes |
| MK540915 | Canada | 1996 | Vulpes vulpes |
| MK540917 | Canada | 1996 | Mephitidae |
| MK540918 | Canada | 1996 | Mephitidae |
| MK540919 | Canada | 1996 | Mephitidae |
| MK540920 | Canada | 1997 | Mephitidae |
| MK540921 | Canada | 1998 | Mephitidae |
| MK540922 | Canada | 1998 | Mephitidae |
| MK540932 | Canada | 1999 | Mephitidae |
| MK540938 | Canada | 2000 | Mephitidae |
| MK540940 | Canada | 2000 | Mephitidae |
| MK540941 | Canada | 2000 | Mephitidae |
| MK540943 | Canada | 2000 | Vulpes vulpes |
| MK540944 | Canada | 2000 | Mephitidae |
| MK540945 | Canada | 2000 | Mephitidae |
| MK540946 | Canada | 2001 | Mephitidae |
| MK540947 | Canada | 2001 | Mephitidae |
| MK540950 | Canada | 2001 | Mephitidae |
| MK540954 | Canada | 2001 | Mephitidae |
| MK540955 | Canada | 2001 | Mephitidae |
| MK540967 | Canada | 2005 | Vulpes vulpes |
| MK540968 | Canada | 2005 | Mephitidae |
| MK540969 | Canada | 2005 | Vulpes vulpes |
| MK540970 | Canada | 2005 | Mephitidae |
| MK540971 | Canada | 2006 | Mephitidae |
| MK540972 | Canada | 2006 | Mephitidae |
| MK540973 | Canada | 2006 | Vulpes vulpes |
| MK540974 | Canada | 2006 | Mephitidae |
| MK540978 | Canada | 2007 | Mephitidae |
| MK540979 | Canada | 2007 | Mephitidae |
| MK540980 | Canada | 2007 | Mephitidae |
| MK540983 | Canada | 2008 | Mephitidae |
| MK540984 | Canada | 2008 | Mephitidae |
| MK540985 | Canada | 2008 | Vulpes vulpes |
| MK540988 | Canada | 2009 | Bos taurus |
| MK540990 | Canada | 2009 | Mephitidae |
| MK540991 | Canada | 2009 | Vulpes vulpes |
| MK540992 | Canada | 2009 | Mephitidae |
| MK540993 | Canada | 2010 | Mephitidae |
| MK540994 | Canada | 2011 | Bos taurus |
| MK540998 | Canada | 2015 | Bos taurus |
| MK541003 | Canada | 2017 | Bos taurus |
| MK541012 | Canada | 2000 | Vulpes vulpes |
| MK567666 | China | 2013 | Canis lupus familiaris |
| MK577649 | China | 2015 | Ovis aries |
| MK920923 | Brazil | 2010 | Potos flavus |
| MN075931 | Thailand | 1999 | Canis lupus familiaris |
| MN175989 | China | 2019 | Bos taurus |
| MN186249 | China | 2018 | Canis lupus familiaris |
| MN186250 | China | 2018 | Canis lupus familiaris |
| MN233900 | USA | 2007 | Vulpes lagopus |
| MN233903 | Canada | 2005 | Vulpes vulpes |
| MN233905 | Canada | 2012 | Canis lupus familiaris |
| MN233911 | Canada | 1996 | Vulpes vulpes |
| MN233917 | Canada | 2003 | Vulpes vulpes |
| MN233923 | Canada | 2004 | Canis lupus familiaris |
| MN233924 | Canada | 2012 | Vulpes vulpes |
| MN233927 | Canada | 2012 | Vulpes vulpes |
| MN233928 | Canada | 2012 | Vulpes vulpes |
| MN233935 | Canada | 2012 | Vulpes vulpes |
| MN233937 | Canada | 2015 | Canis |
| MN233938 | Canada | 2015 | Vulpes vulpes |
| MN233939 | Canada | 2015 | Canis lupus familiaris |
| MN233941 | Canada | 2015 | Vulpes vulpes |
| MN233942 | Canada | 2015 | Vulpes vulpes |
| MN233945 | Canada | 2015 | Vulpes vulpes |
| MN233946 | Canada | 2015 | Felis catus |
| MN233947 | Canada | 1990 | Ursus maritimus |
| MN233948 | Canada | 1990 | Vulpes lagopus |
| MN233951 | Canada | 1993 | Vulpes lagopus |
| MN233952 | Canada | 1993 | Vulpes lagopus |
| MN233956 | Canada | 1993 | Vulpes lagopus |
| MN233960 | Canada | 1998 | Vulpes lagopus |
| MN233963 | Canada | 2001 | Vulpes lagopus |
| MN233964 | Canada | 2005 | Canis lupus familiaris |
| MN233966 | Canada | 2006 | Vulpes lagopus |
| MN233972 | Canada | 2012 | Vulpes lagopus |
| MN233975 | Canada | 1999 | Vulpes lagopus |
| MN233976 | Canada | 2002 | Vulpes vulpes |
| MN233977 | Canada | 2002 | Vulpes lagopus |
| MN233978 | Canada | 2004 | Vulpes lagopus |
| MN233979 | Canada | 2005 | Vulpes vulpes |
| MN233982 | Canada | 2006 | Vulpes lagopus |
| MN233987 | Canada | 2007 | Vulpes lagopus |
| MN233989 | Canada | 2008 | Vulpes vulpes |
| MN233992 | Canada | 2012 | Vulpes lagopus |
| MN233994 | Canada | 2013 | Vulpes lagopus |
| MN233995 | Canada | 2013 | Vulpes lagopus |
| MN233997 | Canada | 2013 | Canis lupus familiaris |
| MN233999 | Canada | 2015 | Vulpes vulpes |
| MN234000 | Canada | 2015 | Vulpes lagopus |
| MN234001 | Canada | 2015 | Vulpes vulpes |
| MN234004 | Canada | 2015 | Vulpes lagopus |
| MN234005 | Canada | 2015 | Canis lupus familiaris |
| MN234006 | Canada | 2015 | Vulpes lagopus |
| MN234007 | Canada | 2016 | Vulpes vulpes |
| MN234008 | Canada | 2017 | Vulpes lagopus |
| MN234009 | Canada | 2017 | Vulpes lagopus |
| MN234010 | Canada | 2017 | Vulpes lagopus |
| MN234011 | Canada | 2017 | Canis lupus familiaris |
| MN234012 | Canada | 2017 | Canis lupus familiaris |
| MN234016 | Canada | 1993 | Vulpes lagopus |
| MN234018 | Canada | 1999 | Vulpes lagopus |
| MN234019 | Canada | 2000 | Canis lupus familiaris |
| MN234024 | Canada | 2003 | Canis lupus familiaris |
| MN234025 | Canada | 2003 | Vulpes vulpes |
| MN234030 | Canada | 2004 | Vulpes vulpes |
| MN234034 | Canada | 2009 | Canis lupus familiaris |
| MN234038 | Canada | 2012 | Vulpes lagopus |
| MN234044 | Canada | 2012 | Vulpes vulpes |
| MN234046 | Canada | 2012 | Vulpes lagopus |
| MN234048 | Canada | 2012 | Vulpes vulpes |
| MN234052 | Canada | 2015 | Vulpes vulpes |
| MN234053 | Canada | 2015 | Canis lupus familiaris |
| MN234054 | Canada | 2015 | Vulpes lagopus |
| MN234055 | Canada | 2017 | Vulpes lagopus |
| MN234056 | Canada | 2017 | Canis lupus familiaris |
| MN418142 | USA | 2018 | Procyon lotor |
| MN418143 | USA | 2008 | Procyon lotor |
| MN418144 | USA | 2017 | Procyon lotor |
| MN418145 | USA | 2017 | Procyon lotor |
| MN418146 | USA | 2017 | Procyon lotor |
| MN418147 | USA | 2017 | Procyon lotor |
| MN418148 | USA | 2017 | Mephitis mephitis |
| MN418149 | USA | 2017 | Vulpes vulpes |
| MN418150 | USA | 2016 | Procyon lotor |
| MN418151 | USA | 2016 | Procyon lotor |
| MN418152 | USA | 2016 | Procyon lotor |
| MN418153 | USA | 2017 | Procyon lotor |
| MN418154 | USA | 2017 | Felis catus |
| MN418155 | USA | 2016 | Procyon lotor |
| MN418156 | USA | 2018 | Procyon lotor |
| MN418157 | USA | 2017 | Procyon lotor |
| MN418158 | USA | 2017 | Urocyon cinereoargenteus |
| MN418159 | USA | 2016 | Procyon lotor |
| MN418160 | USA | 2017 | Procyon lotor |
| MN418161 | USA | 2017 | Lontra canadensis |
| MN418162 | USA | 2017 | Procyon lotor |
| MN418163 | USA | 2016 | Procyon lotor |
| MN418164 | USA | 2009 | Procyon lotor |
| MN418165 | USA | 2017 | Vulpes vulpes |
| MN418166 | USA | 2018 | Canis lupus familiaris |
| MN418167 | USA | 2017 | Procyon lotor |
| MN418168 | USA | 2017 | Mephitis mephitis |
| MN418169 | USA | 2018 | Procyon lotor |
| MN418170 | USA | 2017 | Felis catus |
| MN418171 | USA | 2018 | Procyon lotor |
| MN418172 | USA | 2017 | Mephitis mephitis |
| MN418173 | USA | 2018 | Procyon lotor |
| MN418174 | USA | 2017 | Procyon lotor |
| MN418175 | USA | 2017 | Procyon lotor |
| MN418176 | USA | 2017 | Procyon lotor |
| MN418177 | USA | 2017 | Urocyon cinereoargenteus |
| MN418178 | USA | 2017 | Procyon lotor |
| MN418179 | USA | 2017 | Procyon lotor |
| MN418180 | USA | 2017 | Mephitis mephitis |
| MN418181 | USA | 2016 | Procyon lotor |
| MN418182 | USA | 2016 | Procyon lotor |
| MN418183 | USA | 2017 | Urocyon cinereoargenteus |
| MN418184 | USA | 2017 | Procyon lotor |
| MN857167 | Philippines | 2019 | Canis lupus familiaris |
| MN857168 | Philippines | 2019 | Canis lupus familiaris |
| MN857169 | Philippines | 2019 | Canis lupus familiaris |
| MN857170 | Philippines | 2019 | Canis lupus familiaris |
| MN857171 | Philippines | 2019 | Feliformia |
| MN862283 | USA | 1991 | Procyon lotor |
| MT107888 | Ghana | 2019 | Homo sapiens |
| MT454631 | South Africa | 2015 | Canis lupus familiaris |
| MT454632 | South Africa | 2015 | Proteles cristata |
| MT454633 | South Africa | 2015 | Proteles cristata |
| MT454634 | South Africa | 2015 | Canis lupus familiaris |
| MT454635 | South Africa | 2015 | Canis lupus familiaris |
| MT454636 | South Africa | 2016 | Canis lupus familiaris |
| MT454637 | South Africa | 2016 | Canis lupus familiaris |
| MT454638 | South Africa | 2016 | Otocyon megalotis |
| MT454639 | South Africa | 2016 | Canis lupus familiaris |
| MT454640 | South Africa | 2016 | Otocyon megalotis |
| MT454641 | South Africa | 2016 | Canis lupus familiaris |
| MT454642 | South Africa | 2016 | Proteles cristata |
| MT454643 | South Africa | 2017 | Canis lupus familiaris |
| MT454644 | South Africa | 2017 | Canis lupus familiaris |
| MT454645 | South Africa | 2017 | Canis lupus familiaris |
| MT454646 | South Africa | 2017 | Canis mesomelas |
| MT454647 | South Africa | 2017 | Canis mesomelas |
| MT454648 | South Africa | 2017 | Canis mesomelas |
| MT454649 | South Africa | 2017 | Canis mesomelas |
| MT454650 | South Africa | 2017 | Otocyon megalotis |
| MT454651 | South Africa | 2017 | Canis mesomelas |
| MT454652 | South Africa | 2017 | Canis mesomelas |
| MT454653 | South Africa | 2017 | Canis mesomelas |
| MT454654 | South Africa | 2017 | Canis mesomelas |
| MT895954 | China | 2019 | Mus musculus |
| MT895955 | China | 2019 | Mus musculus |
| MT895956 | China | 2019 | Mus musculus |
| MT895957 | China | 2019 | Mus musculus |
| MT895958 | China | 2019 | Mus musculus |
| MT895959 | China | 2019 | Mus musculus |
| MT895960 | China | 2019 | Mus musculus |
| MT895961 | China | 2019 | Mus musculus |
| MT895962 | China | 2019 | Mus musculus |
| MT895963 | China | 2019 | Mus musculus |
| MT895965 | China | 2019 | Mus musculus |
| MT895966 | China | 2019 | Mus musculus |
| MT895967 | China | 2019 | Mus musculus |
| MT895968 | China | 2019 | Mus musculus |
| MW506937 | Costa Rica | 2018 | Bos taurus |
| MW506938 | Costa Rica | 2018 | Bos taurus |
| MW506939 | Costa Rica | 2018 | Bos taurus |
| MW506940 | Costa Rica | 2018 | Bos taurus |
| MZ494188 | USA | 2020 | Chiroptera |
| OK135144 | Liberia | 2017 | Felis catus |
| OK135145 | Liberia | 2017 | Canis lupus familiaris |
| OK135146 | Liberia | 2018 | Canis lupus familiaris |
| OK135147 | Liberia | 2018 | Canis lupus familiaris |
| OK135148 | Liberia | 2018 | Canis lupus familiaris |
| OK317992 | Congo | 2017 | Canis lupus familiaris |
| OK317993 | Congo | 2018 | Canis lupus familiaris |
| OK317994 | Congo | 2018 | Canis lupus familiaris |
| OK317995 | Congo | 2017 | Canis lupus familiaris |
| OK317996 | Congo | 2018 | Canis lupus familiaris |
| OK317997 | Congo | 2017 | Felis catus |
| OK317998 | Congo | 2017 | Canis lupus familiaris |
| OK317999 | Congo | 2018 | Canis lupus familiaris |
| OU524425 | Canada | 1986 | Eptesicus fuscus |

**Table S2 RABV Temporal signal comparison.**

| **Molecular clock model** | **Tip dates** | **Log marginal likelihood** | |
| --- | --- | --- | --- |
|  |  | **N** | **L** |
| Strict clock | Sampling dates | -33965.64 | -154303.72 |
| Strict clock | No sampling dates | -34375.68 | -155817.85 |
| Uncorrelated relaxed | Sampling dates | -33932.55 | -155574.79 |
| Uncorrelated relaxed clock | No sampling dates | -2.59E+07 | -154852.66 |

Smaller value of Log marginal likelihood indicated better model.

**Table S3 RABV prior model comparison.**

| **Molecular clock model** | **Log marginal likelihood** | |
| --- | --- | --- |
|  | **N** | **L** |
| Coalescent bayesian skyline clock | -33902.60  -33902.6 | -154778.18 |
| Coalescent constant size | -33966.74 | -155122.23 |

Smaller value of Log marginal likelihood indicated better model.

**Table S4 BSSVS analysis data of N genes for regions.**

| **From** | **To** | **Bayes** | **Posterior** | **Migration** |
| --- | --- | --- | --- | --- |
|  |  | **factor** | **probability** | **rate** |
| Africa | Asia | 0.85 | 0.14 | 0.95 |
| Africa | Europe | 0.87 | 0.14 | 0.96 |
| Africa | NA-M | 0.91 | 0.15 | 0.94 |
| Africa | NA-N | 1.02 | 0.16 | 0.94 |
| Africa | NA-S | 0.91 | 0.15 | 0.93 |
| Africa | SA | 0.80 | 0.13 | 0.95 |
| Asia | Africa | 1.12 | 0.18 | 0.92 |
| Asia | Europe | 0.85 | 0.14 | 0.93 |
| Asia | NA-M | 0.96 | 0.15 | 0.92 |
| Asia | NA-N | 1.60 | 0.23 | 0.90 |
| Asia | NA-S | 2.35 | 0.31 | 0.91 |
| Asia | SA | 0.77 | 0.13 | 0.94 |
| Europe | Africa | 8.54 | 0.62 | 0.92 |
| Europe | Asia | 47475.67 | 1.00 | 2.14 |
| Europe | NA-M | 0.84 | 0.14 | 0.96 |
| Europe | NA-N | 2.21 | 0.30 | 1.17 |
| Europe | NA-S | 9.65 | 0.65 | 0.96 |
| Europe | SA | 0.46 | 0.08 | 0.97 |
| NA-M | Africa | 1.07 | 0.17 | 0.92 |
| NA-M | Asia | 0.96 | 0.15 | 0.91 |
| NA-M | Europe | 1.32 | 0.20 | 0.91 |
| NA-M | NA-N | 2.35 | 0.31 | 0.91 |
| NA-M | NA-S | 1.22 | 0.19 | 0.95 |
| NA-M | SA | 0.73 | 0.12 | 0.95 |
| NA-N | Africa | 0.51 | 0.09 | 0.93 |
| NA-N | Asia | 0.79 | 0.13 | 0.95 |
| NA-N | Europe | 1.31 | 0.20 | 1.53 |
| NA-N | NA-M | 13.50 | 0.72 | 0.96 |
| NA-N | NA-S | 0.85 | 0.14 | 0.94 |
| NA-N | SA | 1351.32 | 1.00 | 0.95 |
| NA-S | Africa | 4.16 | 0.44 | 0.94 |
| NA-S | Asia | 0.97 | 0.15 | 0.95 |
| NA-S | Europe | 11864.96 | 1.00 | 0.93 |
| NA-S | NA-M | 0.74 | 0.12 | 0.80 |
| NA-S | NA-N | 1.43 | 0.21 | 0.93 |
| NA-S | SA | 0.51 | 0.09 | 1.97 |
| SA | Africa | 1.65 | 0.24 | 0.89 |
| SA | Asia | 1.37 | 0.21 | 0.92 |
| SA | Europe | 1.57 | 0.23 | 0.90 |
| SA | NA-M | 1.70 | 0.24 | 0.93 |
| SA | NA-N | 1.14 | 0.18 | 0.89 |
| SA | NA-S | 1.68 | 0.24 | 0.93 |

Highlight lines were selected for sufficient bayes factor (BF > 3) and posterior probability (PP > 0.5) support.

**Table S5 BSSVS analysis data of N gene for species.**

| **From** | **To** | **Bayes** | **Posterior** | **Migration** |
| --- | --- | --- | --- | --- |
|  |  | **factor** | **probability** | **rate** |
| Bovidae | Cervidae | 0.53 | 0.06 | 0.99 |
| Bovidae | Equidae | 1.27 | 0.13 | 0.94 |
| Bovidae | Felidae | 0.50 | 0.06 | 0.98 |
| Bovidae | Hominidae | 0.38 | 0.04 | 0.98 |
| Bovidae | Mephitidae | 0.47 | 0.05 | 1.00 |
| Bovidae | Procyonidae | 1.91 | 0.19 | 0.91 |
| Bovidae | Ursidae | 1.65 | 0.17 | 0.88 |
| Bovidae | Vespertilionidae | 19.83 | 0.71 | 0.79 |
| Canidae | Bovidae | 335572.00 | 1.00 | 0.96 |
| Canidae | Cervidae | 271.13 | 0.97 | 0.24 |
| Canidae | Equidae | 5.90 | 0.42 | 0.65 |
| Canidae | Felidae | 7.31 | 0.47 | 0.61 |
| Canidae | Hominidae | 335572.00 | 1.00 | 0.94 |
| Canidae | Mephitidae | 136.30 | 0.94 | 0.72 |
| Canidae | Procyonidae | 2.09 | 0.20 | 0.86 |
| Canidae | Ursidae | 7.93 | 0.49 | 0.58 |
| Canidae | Vespertilionidae | 0.23 | 0.03 | 1.00 |
| Cervidae | Bovidae | 0.67 | 0.07 | 0.97 |
| Cervidae | Canidae | 0.71 | 0.08 | 0.95 |
| Cervidae | Equidae | 1.17 | 0.12 | 0.95 |
| Cervidae | Felidae | 0.77 | 0.08 | 0.95 |
| Cervidae | Hominidae | 0.61 | 0.07 | 0.98 |
| Cervidae | Mephitidae | 0.77 | 0.09 | 0.93 |
| Cervidae | Procyonidae | 0.98 | 0.11 | 0.97 |
| Cervidae | Ursidae | 1.33 | 0.14 | 0.91 |
| Cervidae | Vespertilionidae | 1.17 | 0.12 | 0.93 |
| Equidae | Bovidae | 1.12 | 0.12 | 0.94 |
| Equidae | Canidae | 1.24 | 0.13 | 0.89 |
| Equidae | Cervidae | 0.79 | 0.09 | 0.95 |
| Equidae | Felidae | 0.99 | 0.11 | 0.92 |
| Equidae | Hominidae | 0.87 | 0.10 | 0.95 |
| Equidae | Mephitidae | 1.21 | 0.13 | 0.92 |
| Equidae | Procyonidae | 1.58 | 0.16 | 0.88 |
| Equidae | Ursidae | 1.35 | 0.14 | 0.90 |
| Equidae | Vespertilionidae | 2.07 | 0.20 | 0.86 |
| Felidae | Bovidae | 0.61 | 0.07 | 0.96 |
| Felidae | Canidae | 0.62 | 0.07 | 0.94 |
| Felidae | Cervidae | 0.55 | 0.06 | 0.96 |
| Felidae | Equidae | 0.75 | 0.08 | 0.95 |
| Felidae | Hominidae | 0.50 | 0.06 | 0.96 |
| Felidae | Mephitidae | 0.72 | 0.08 | 0.95 |
| Felidae | Procyonidae | 1.28 | 0.13 | 0.99 |
| Felidae | Ursidae | 0.73 | 0.08 | 0.95 |
| Felidae | Vespertilionidae | 1.25 | 0.13 | 0.94 |
| Hominidae | Bovidae | 0.57 | 0.06 | 0.95 |
| Hominidae | Canidae | 0.80 | 0.09 | 0.95 |
| Hominidae | Cervidae | 0.47 | 0.05 | 0.97 |
| Hominidae | Equidae | 2.06 | 0.20 | 0.89 |
| Hominidae | Felidae | 4.66 | 0.36 | 0.79 |
| Hominidae | Mephitidae | 0.72 | 0.08 | 0.97 |
| Hominidae | Procyonidae | 1.93 | 0.19 | 0.89 |
| Hominidae | Ursidae | 2.99 | 0.27 | 0.85 |
| Hominidae | Vespertilionidae | 0.89 | 0.10 | 0.92 |
| Mephitidae | Bovidae | 344.96 | 0.98 | 0.88 |
| Mephitidae | Canidae | 335572.00 | 1.00 | 2.96 |
| Mephitidae | Cervidae | 0.15 | 0.02 | 0.98 |
| Mephitidae | Equidae | 1.18 | 0.12 | 0.92 |
| Mephitidae | Felidae | 0.38 | 0.04 | 0.97 |
| Mephitidae | Hominidae | 0.20 | 0.02 | 0.98 |
| Mephitidae | Procyonidae | 1.69 | 0.17 | 0.93 |
| Mephitidae | Ursidae | 0.44 | 0.05 | 0.97 |
| Mephitidae | Vespertilionidae | 0.31 | 0.04 | 0.99 |
| Procyonidae | Bovidae | 3.41 | 0.29 | 0.84 |
| Procyonidae | Canidae | 335572.00 | 1.00 | 1.57 |
| Procyonidae | Cervidae | 0.14 | 0.02 | 0.99 |
| Procyonidae | Equidae | 104.59 | 0.93 | 0.43 |
| Procyonidae | Felidae | 335572.00 | 1.00 | 1.63 |
| Procyonidae | Hominidae | 0.12 | 0.01 | 1.00 |
| Procyonidae | Mephitidae | 335572.00 | 1.00 | 6.97 |
| Procyonidae | Ursidae | 0.14 | 0.02 | 0.99 |
| Procyonidae | Vespertilionidae | 0.18 | 0.02 | 1.01 |
| Ursidae | Bovidae | 0.66 | 0.07 | 0.96 |
| Ursidae | Canidae | 0.72 | 0.08 | 0.97 |
| Ursidae | Cervidae | 0.66 | 0.07 | 0.98 |
| Ursidae | Equidae | 0.84 | 0.09 | 0.95 |
| Ursidae | Felidae | 0.68 | 0.08 | 0.95 |
| Ursidae | Hominidae | 0.66 | 0.07 | 0.97 |
| Ursidae | Mephitidae | 0.72 | 0.08 | 0.97 |
| Ursidae | Procyonidae | 0.85 | 0.09 | 0.96 |
| Ursidae | Vespertilionidae | 1.21 | 0.13 | 0.90 |
| Vespertilionidae | Bovidae | 0.63 | 0.07 | 0.95 |
| Vespertilionidae | Canidae | 7795.91 | 1.00 | 0.69 |
| Vespertilionidae | Cervidae | 0.09 | 0.01 | 0.98 |
| Vespertilionidae | Equidae | 0.09 | 0.01 | 1.02 |
| Vespertilionidae | Felidae | 15.73 | 0.65 | 0.43 |
| Vespertilionidae | Hominidae | 0.16 | 0.02 | 0.99 |
| Vespertilionidae | Mephitidae | 163.19 | 0.95 | 0.50 |
| Vespertilionidae | Procyonidae | 3.30 | 0.28 | 0.80 |
| Vespertilionidae | Ursidae | 0.08 | 0.01 | 0.98 |

Highlight lines were selected for sufficient bayes factor (BF > 3) and posterior probability (PP > 0.5) support.

**Table S6 BSSVS analysis data of L gene for regions.**

| **From** | **To** | **Bayes** | **Posterior** | **Migration** |
| --- | --- | --- | --- | --- |
|  |  | **factor** | **probability** | **rate** |
| Africa | Asia | 0.86 | 0.14 | 0.95 |
| Africa | Europe | 0.85 | 0.14 | 0.91 |
| Africa | NA-M | 0.93 | 0.15 | 0.95 |
| Africa | NA-N | 0.97 | 0.16 | 0.94 |
| Africa | NA-S | 0.93 | 0.15 | 0.95 |
| Africa | SA | 0.82 | 0.13 | 0.94 |
| Asia | Africa | 1.02 | 0.16 | 0.91 |
| Asia | Europe | 0.81 | 0.13 | 0.94 |
| Asia | NA-M | 0.90 | 0.15 | 0.92 |
| Asia | NA-N | 1.53 | 0.22 | 0.87 |
| Asia | NA-S | 2.32 | 0.31 | 0.91 |
| Asia | SA | 0.69 | 0.12 | 0.93 |
| Europe | Africa | 7.99 | 0.60 | 0.90 |
| Europe | Asia | 47496.77 | 1.00 | 2.03 |
| Europe | NA-M | 0.71 | 0.12 | 0.95 |
| Europe | NA-N | 1.98 | 0.27 | 1.12 |
| Europe | NA-S | 8.86 | 0.63 | 0.93 |
| Europe | SA | 0.47 | 0.08 | 0.96 |
| NA-M | Africa | 1.20 | 0.18 | 0.88 |
| NA-M | Asia | 1.05 | 0.17 | 0.92 |
| NA-M | Europe | 1.22 | 0.19 | 0.90 |
| NA-M | NA-N | 1.64 | 0.24 | 0.91 |
| NA-M | NA-S | 1.19 | 0.18 | 0.94 |
| NA-M | SA | 0.83 | 0.14 | 0.93 |
| NA-N | Africa | 0.48 | 0.08 | 0.91 |
| NA-N | Asia | 0.79 | 0.13 | 0.97 |
| NA-N | Europe | 1.32 | 0.20 | 2.08 |
| NA-N | NA-M | 8.73 | 0.62 | 0.96 |
| NA-N | NA-S | 0.95 | 0.15 | 0.94 |
| NA-N | SA | 11870.24 | 1.00 | 0.94 |
| NA-S | Africa | 4.44 | 0.46 | 0.95 |
| NA-S | Asia | 0.95 | 0.15 | 0.94 |
| NA-S | Europe | 47496.77 | 1.00 | 0.92 |
| NA-S | NA-M | 0.73 | 0.12 | 0.77 |
| NA-S | NA-N | 1.66 | 0.24 | 0.92 |
| NA-S | SA | 0.50 | 0.09 | 1.77 |
| SA | Africa | 1.52 | 0.22 | 0.87 |
| SA | Asia | 1.29 | 0.20 | 0.91 |
| SA | Europe | 1.51 | 0.22 | 0.90 |
| SA | NA-M | 2.95 | 0.36 | 0.92 |
| SA | NA-N | 0.95 | 0.15 | 0.90 |
| SA | NA-S | 1.54 | 0.23 | 0.92 |

Highlight lines were selected for sufficient bayes factor (BF > 3) and posterior probability (PP > 0.5) support.

**Table S7 BSSVS analysis data of L gene for species.**

| **From** | **To** | **Bayes** | **Posterior probability** | **Migration rate** |
| --- | --- | --- | --- | --- |
|  |  | **factor** |  |  |
| Bovidae | Canidae | 0.27 | 0.03 | 0.97 |
| Bovidae | Cervidae | 0.22 | 0.03 | 0.98 |
| Bovidae | Equidae | 0.96 | 0.10 | 0.89 |
| Bovidae | Felidae | 0.18 | 0.02 | 1.01 |
| Bovidae | Hominidae | 0.35 | 0.04 | 0.97 |
| Bovidae | Mephitidae | 0.43 | 0.05 | 0.96 |
| Bovidae | Procyonidae | 1.93 | 0.19 | 0.93 |
| Bovidae | Ursidae | 0.22 | 0.03 | 1.00 |
| Bovidae | Vespertilionidae | 107.92 | 0.93 | 0.71 |
| Canidae | Bovidae | 74597.33 | 1.00 | 1.05 |
| Canidae | Cervidae | 1214.76 | 0.99 | 0.18 |
| Canidae | Equidae | 3.07 | 0.27 | 0.75 |
| Canidae | Felidae | 74597.33 | 1.00 | 0.33 |
| Canidae | Hominidae | 74597.33 | 1.00 | 0.77 |
| Canidae | Mephitidae | 8281.23 | 1.00 | 0.54 |
| Canidae | Procyonidae | 0.45 | 0.05 | 0.97 |
| Canidae | Ursidae | 924.29 | 0.99 | 0.18 |
| Canidae | Vespertilionidae | 0.83 | 0.09 | 0.94 |
| Cervidae | Bovidae | 0.60 | 0.07 | 0.98 |
| Cervidae | Canidae | 0.61 | 0.07 | 0.97 |
| Cervidae | Equidae | 0.94 | 0.10 | 0.93 |
| Cervidae | Felidae | 0.57 | 0.06 | 0.93 |
| Cervidae | Hominidae | 0.57 | 0.06 | 0.99 |
| Cervidae | Mephitidae | 0.61 | 0.07 | 0.96 |
| Cervidae | Procyonidae | 0.99 | 0.11 | 0.93 |
| Cervidae | Ursidae | 0.56 | 0.06 | 0.96 |
| Cervidae | Vespertilionidae | 0.78 | 0.09 | 0.93 |
| Equidae | Bovidae | 1.10 | 0.12 | 0.90 |
| Equidae | Canidae | 1.24 | 0.13 | 0.93 |
| Equidae | Cervidae | 1.00 | 0.11 | 0.88 |
| Equidae | Felidae | 0.81 | 0.09 | 0.98 |
| Equidae | Hominidae | 0.95 | 0.10 | 0.89 |
| Equidae | Mephitidae | 1.29 | 0.14 | 0.87 |
| Equidae | Procyonidae | 2.17 | 0.21 | 0.86 |
| Equidae | Ursidae | 0.99 | 0.11 | 0.91 |
| Equidae | Vespertilionidae | 1.26 | 0.13 | 0.87 |
| Felidae | Bovidae | 0.51 | 0.06 | 0.98 |
| Felidae | Canidae | 0.55 | 0.06 | 0.95 |
| Felidae | Cervidae | 0.46 | 0.05 | 0.99 |
| Felidae | Equidae | 1.23 | 0.13 | 0.91 |
| Felidae | Hominidae | 0.52 | 0.06 | 0.98 |
| Felidae | Mephitidae | 0.62 | 0.07 | 0.98 |
| Felidae | Procyonidae | 1.77 | 0.18 | 0.94 |
| Felidae | Ursidae | 0.46 | 0.05 | 0.96 |
| Felidae | Vespertilionidae | 0.72 | 0.08 | 0.94 |
| Hominidae | Bovidae | 0.71 | 0.08 | 0.91 |
| Hominidae | Canidae | 1.10 | 0.12 | 0.91 |
| Hominidae | Cervidae | 0.55 | 0.06 | 0.94 |
| Hominidae | Equidae | 2.62 | 0.24 | 0.83 |
| Hominidae | Felidae | 0.56 | 0.06 | 0.97 |
| Hominidae | Mephitidae | 1.00 | 0.11 | 0.91 |
| Hominidae | Procyonidae | 4.66 | 0.36 | 0.76 |
| Hominidae | Ursidae | 0.61 | 0.07 | 0.97 |
| Hominidae | Vespertilionidae | 0.81 | 0.09 | 0.94 |
| Mephitidae | Bovidae | 8.62 | 0.51 | 0.69 |
| Mephitidae | Canidae | 74597.33 | 1.00 | 2.53 |
| Mephitidae | Cervidae | 0.11 | 0.01 | 1.04 |
| Mephitidae | Equidae | 4.31 | 0.34 | 0.77 |
| Mephitidae | Felidae | 0.15 | 0.02 | 0.97 |
| Mephitidae | Hominidae | 0.18 | 0.02 | 0.93 |
| Mephitidae | Procyonidae | 1.43 | 0.15 | 0.91 |
| Mephitidae | Ursidae | 0.10 | 0.01 | 1.03 |
| Mephitidae | Vespertilionidae | 0.47 | 0.05 | 0.95 |
| Procyonidae | Bovidae | 74597.33 | 1.00 | 0.82 |
| Procyonidae | Canidae | 74597.33 | 1.00 | 1.83 |
| Procyonidae | Cervidae | 0.12 | 0.01 | 1.00 |
| Procyonidae | Equidae | 17.63 | 0.68 | 0.56 |
| Procyonidae | Felidae | 74597.33 | 1.00 | 1.31 |
| Procyonidae | Hominidae | 0.12 | 0.01 | 1.00 |
| Procyonidae | Mephitidae | 74597.33 | 1.00 | 8.02 |
| Procyonidae | Ursidae | 0.11 | 0.01 | 0.98 |
| Procyonidae | Vespertilionidae | 0.15 | 0.02 | 1.01 |
| Ursidae | Bovidae | 0.54 | 0.06 | 0.95 |
| Ursidae | Canidae | 0.52 | 0.06 | 0.96 |
| Ursidae | Cervidae | 0.53 | 0.06 | 0.95 |
| Ursidae | Equidae | 0.78 | 0.09 | 0.95 |
| Ursidae | Felidae | 0.56 | 0.06 | 0.93 |
| Ursidae | Hominidae | 0.52 | 0.06 | 1.00 |
| Ursidae | Mephitidae | 0.62 | 0.07 | 0.92 |
| Ursidae | Procyonidae | 1.00 | 0.11 | 0.91 |
| Ursidae | Vespertilionidae | 0.59 | 0.07 | 0.96 |
| Vespertilionidae | Bovidae | 0.18 | 0.02 | 0.99 |
| Vespertilionidae | Canidae | 1613.58 | 0.99 | 0.53 |
| Vespertilionidae | Cervidae | 0.07 | 0.01 | 0.99 |
| Vespertilionidae | Equidae | 0.06 | 0.01 | 0.99 |
| Vespertilionidae | Felidae | 5.22 | 0.39 | 0.66 |
| Vespertilionidae | Hominidae | 0.09 | 0.01 | 0.99 |
| Vespertilionidae | Mephitidae | 820.67 | 0.99 | 0.43 |
| Vespertilionidae | Procyonidae | 1.35 | 0.14 | 0.88 |
| Vespertilionidae | Ursidae | 0.06 | 0.01 | 0.99 |

Highlight lines were selected for sufficient bayes factor (BF > 3) and posterior probability (PP > 0.5) support.


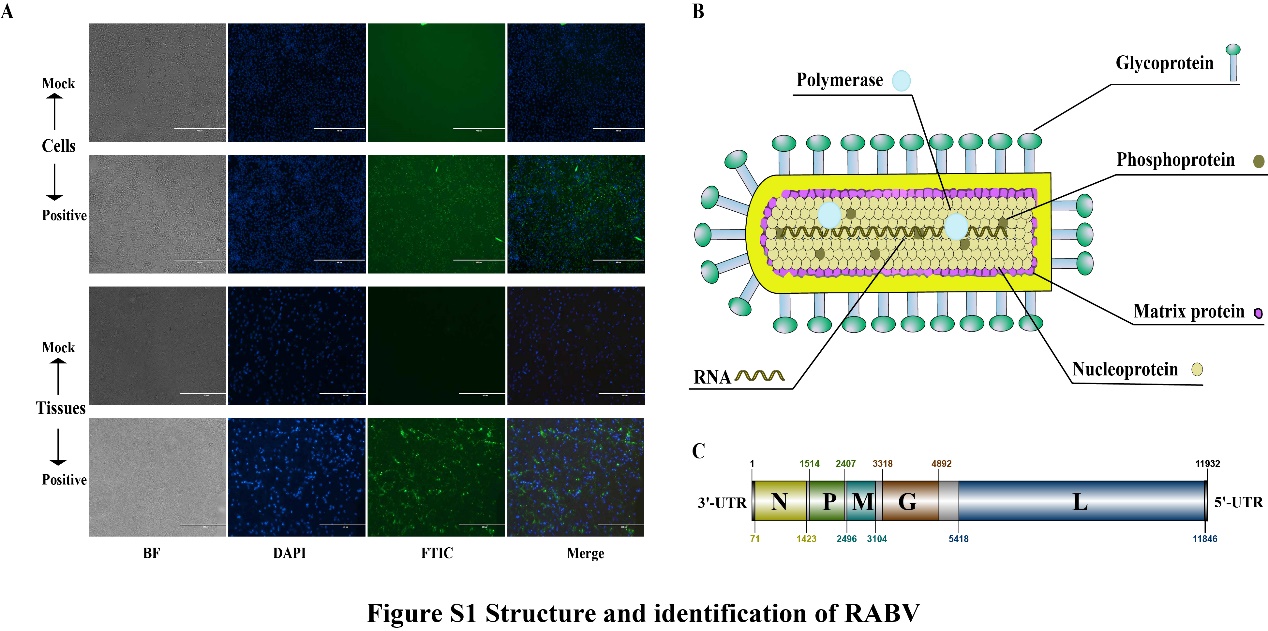


**Figure S1 Structure and identification of RABV.**

1. NA cells and hippocampal neurons of a laboratory mouse infected with GD-SH strain.
2. The virion of rabies virus is bullet-shaped, with five viral proteins: the nucleoprotein (N), phosphoprotein (P), matrix protein (M), glycoprotein (G) and large RNA polymerase protein (L).
3. Rabies virus is a negative-strand RNA virus with a length of about 12kb, encoding N, P, M, G and L from3’ end to 5’ end.


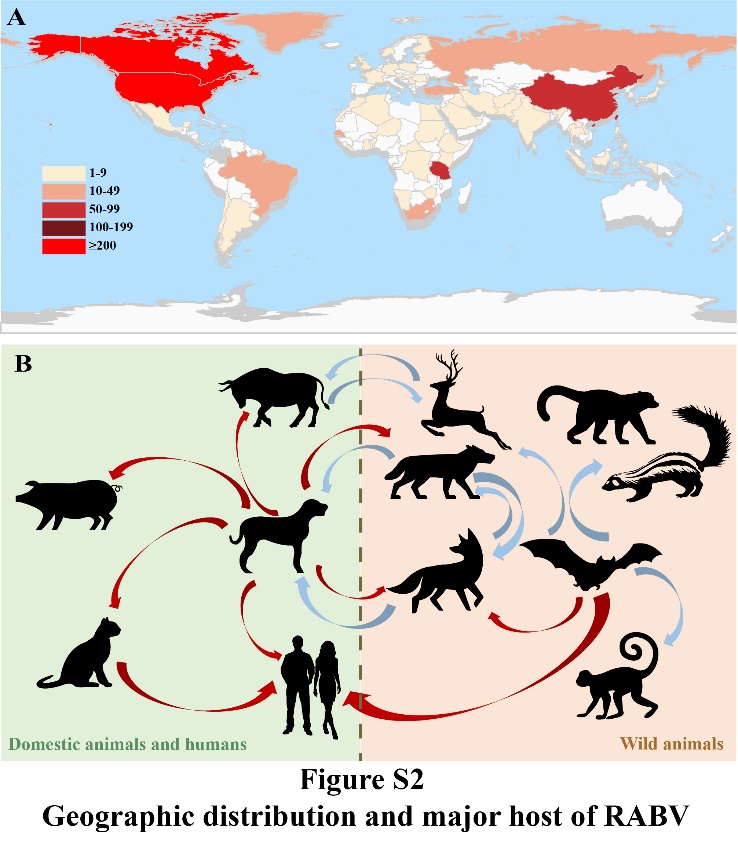


**Figure S2 Geographic distribution and major host of RABV.**

The green area represents main domestic animals and humans; the orange area represents main wild animals.

1. Geographic distribution of the RABV full-length genomes.
2. Major host of RABV; the red lines represent definite transmission routes, grey lines represent unproven transmission routes.


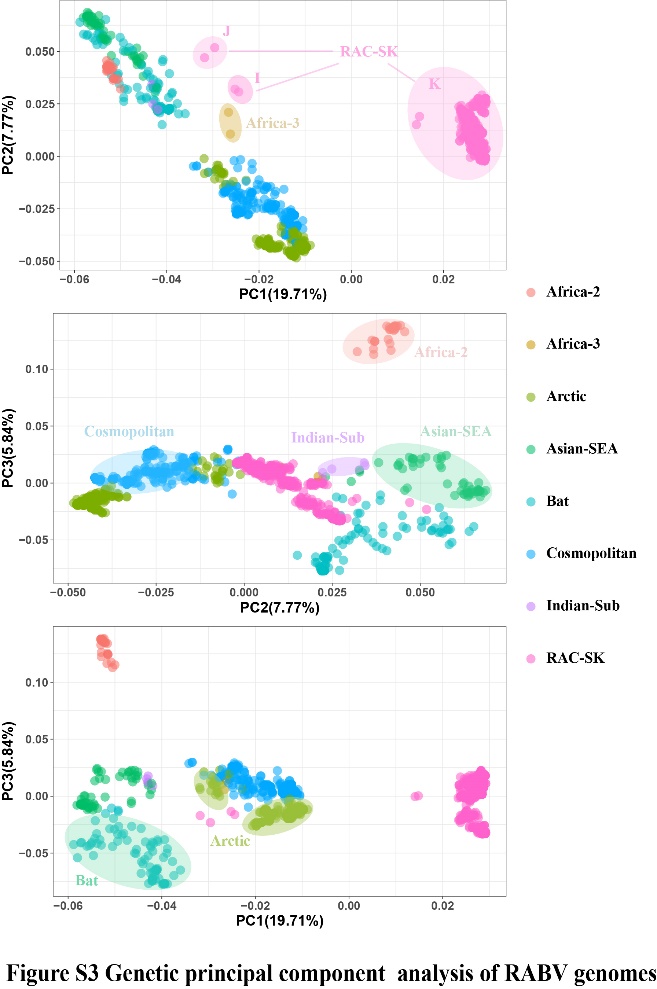


**Figure S3 Principal component analysis.**

Using pairwise combinations between 1, 2, and 3 to show different clustering perspectives. Different colours represent different lineages. There are the combination of PC1 and PC2 in the top figure, the combination of PC2 and PC3 in the middle figure, and the combination of PC2 and PC3 in the bottom figure.


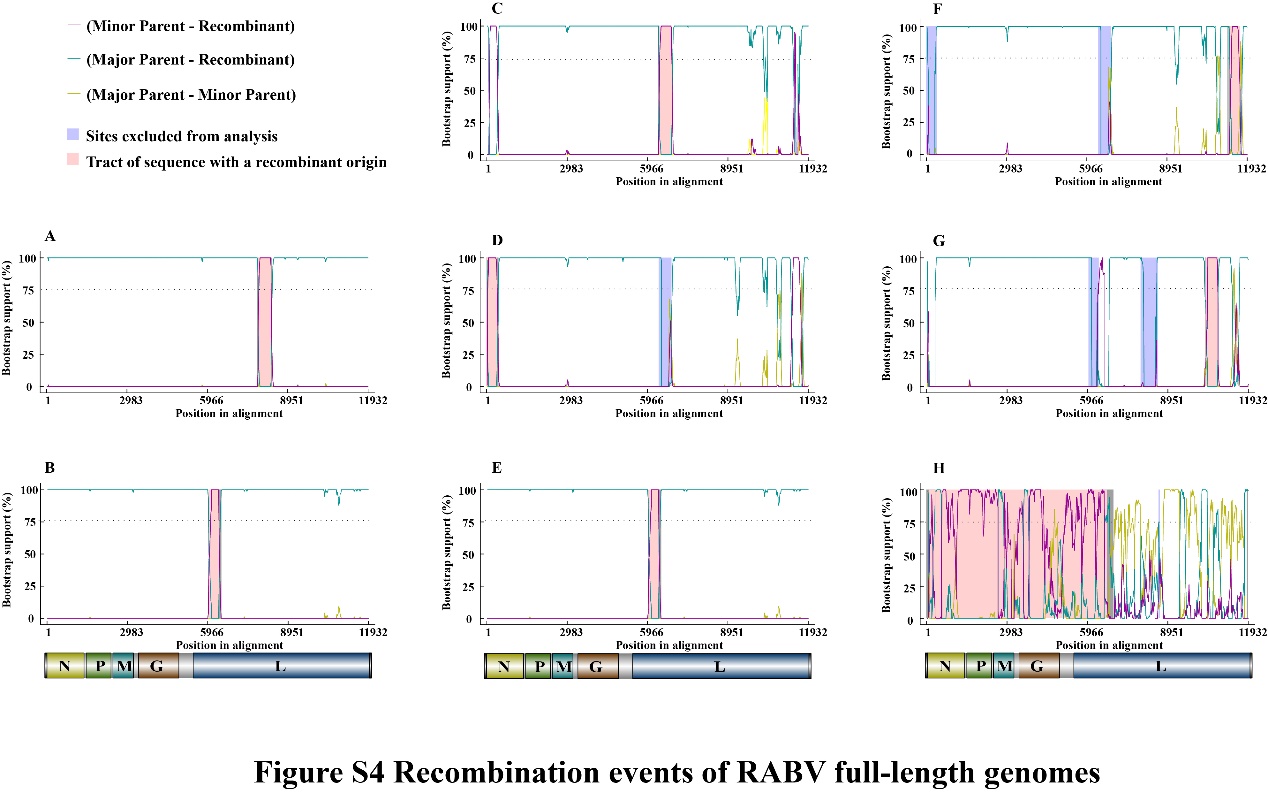


**Figure S4 Recombination events of RABV full-length genomes.**

A, B, C, D, E, F and G correspond to Table 2.


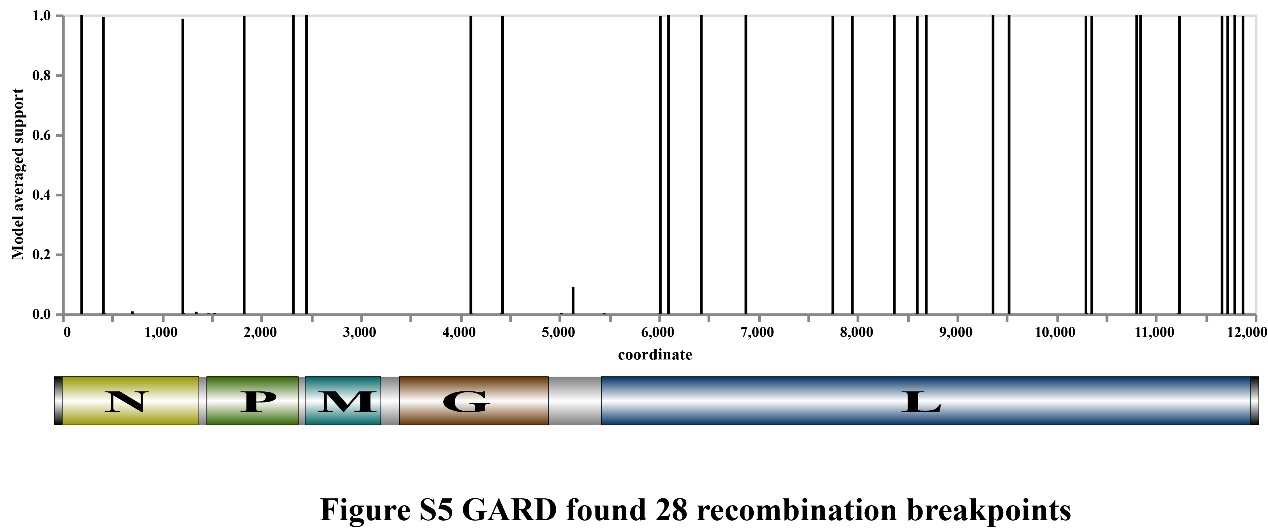


**Figure S5 GARD found 28 recombination breakpoints.**

There 3 breakpoints in the region of N gene and 20 breakpoints in L gene.


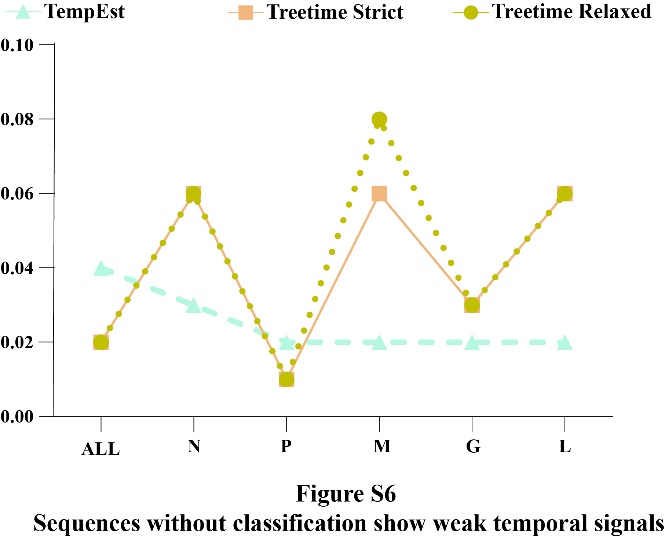


**Figure S6 Sequences without classification show weak temporal signals.**

Three different models estimate temporal signal to get *R^2^*.


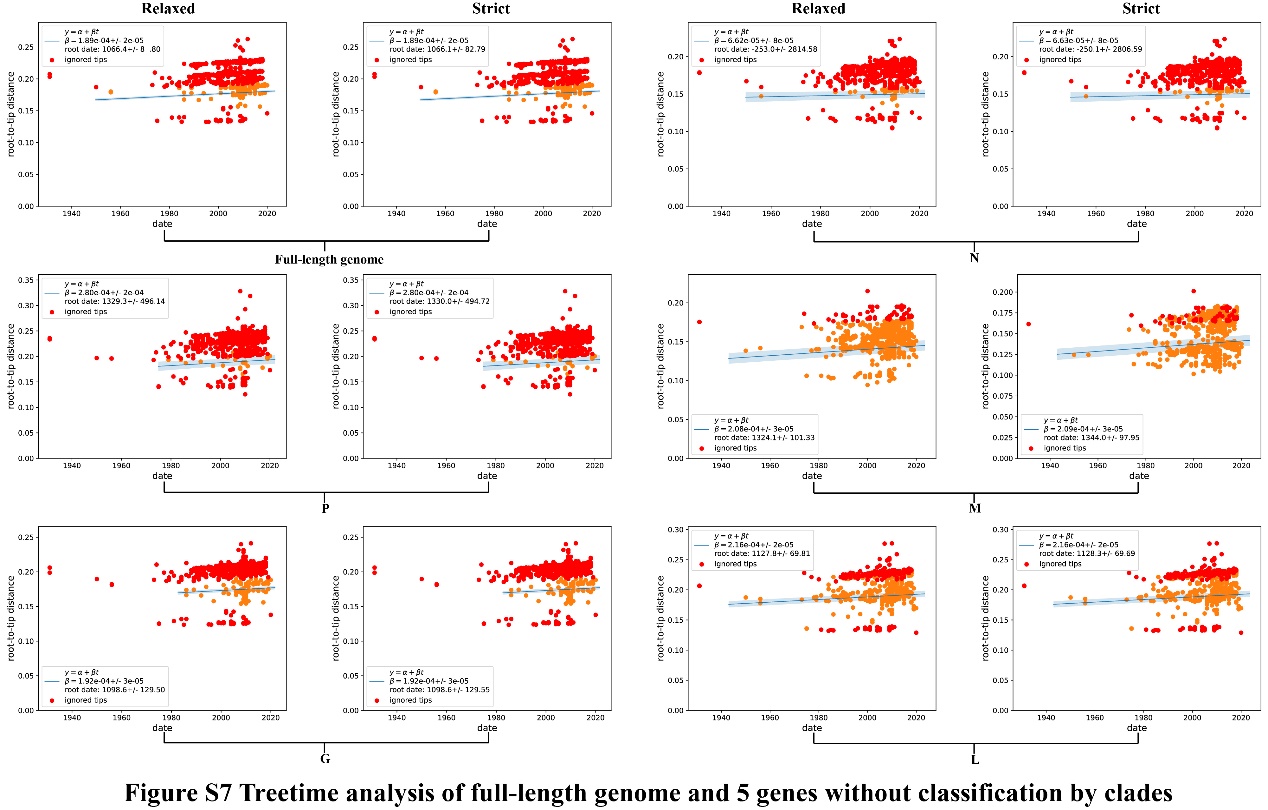


**Figure S7 Treetime analysis of full-length genome and 5 genes without classification by lineages.**

The red plots represent sequences that deviate from the molecular clock model and are ignored when evaluating the temporal signal.

The root-to-top regressions of full-length genome and 5 genes, N, P, M, G and L, show a lot of sequences deviating from the molecular clock model.


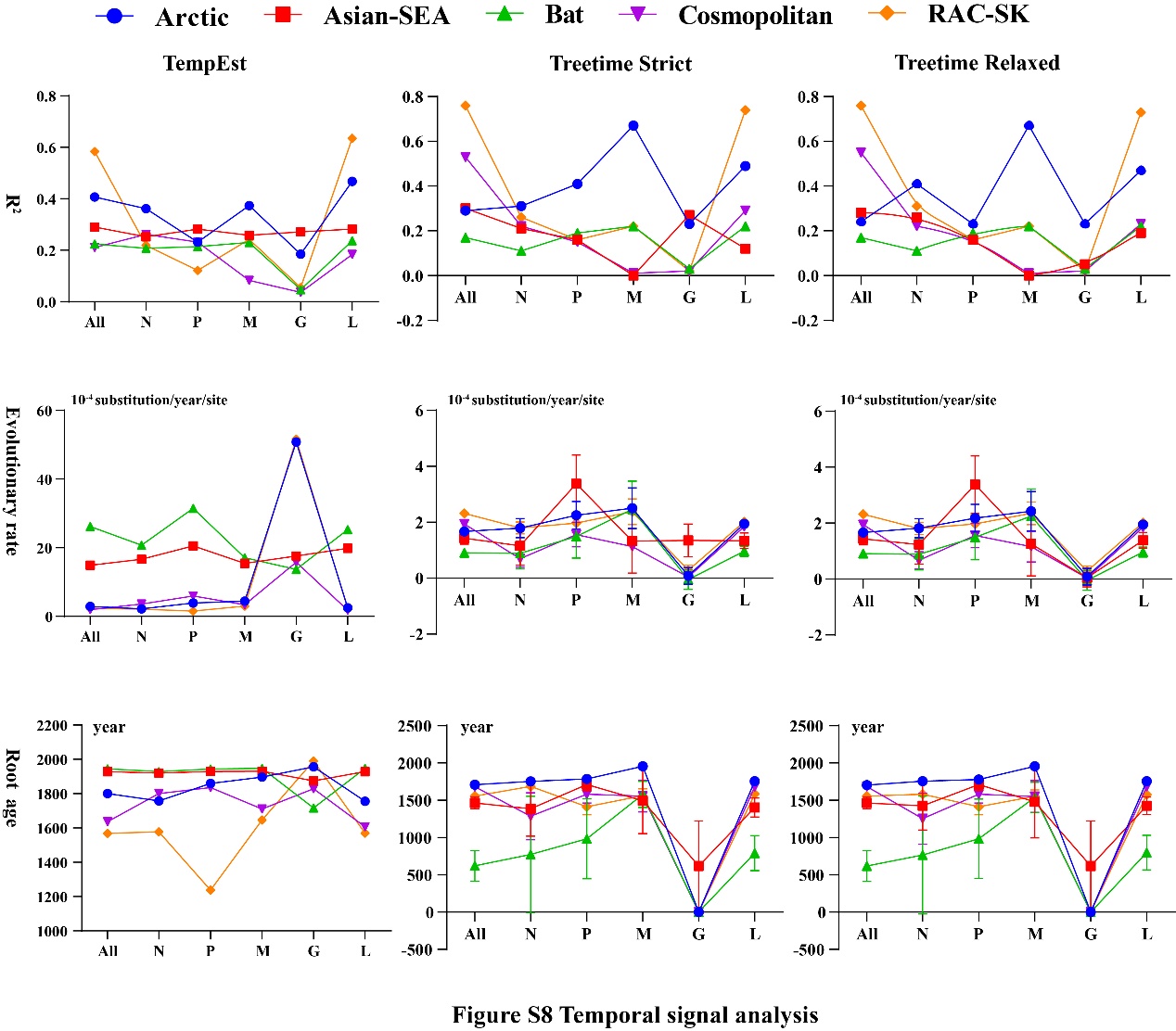


**Figure S8 Temporal signal analysis.**

There are the figures of root-to-tip regression of full-length genomes, N genes, P genes, M genes, G genes and L genes with relaxed and strict model by Treetime.


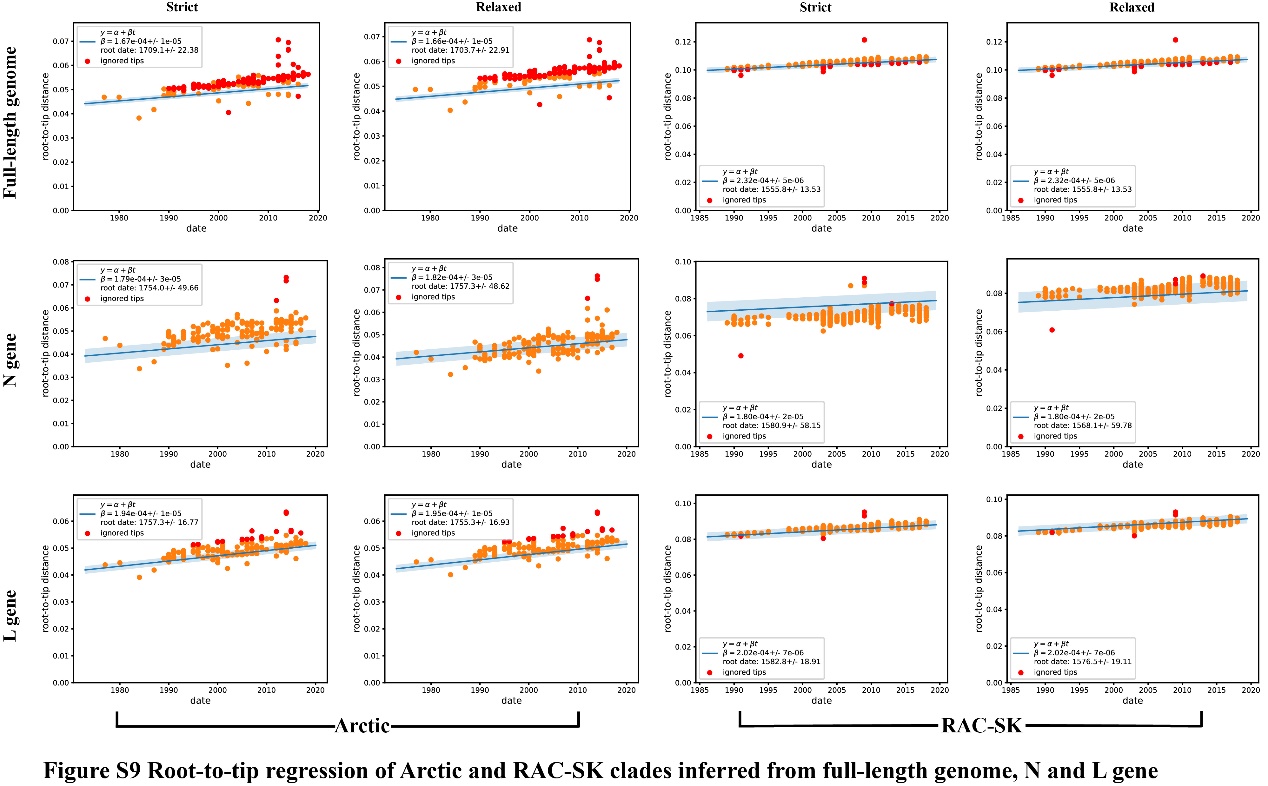


**Figure S9 Root-to-tip regression of Arctic and RAC-SK groups inferred from full-length genome, N and L gene.**

The red plots represent ignored sequences without temporal signal.

Compared to the N and L genes, there are a lot of red plots in the root-to-top repression of full-length genome.


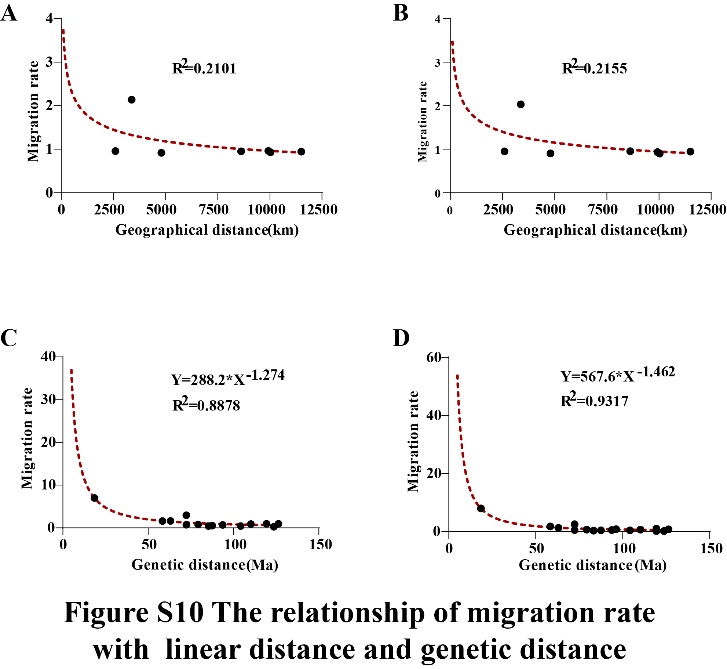


**Figure S10 The relationship of** **migration rate with linear distance and genetic distance.**

1. The relationship between migration rate and linear distance for N gene.
2. The relationship between migration rate and linear distance for L gene.
3. The relationship between migration rate and genetic distance for N gene.
4. The relationship between migration rate and genetic distance for L gene.


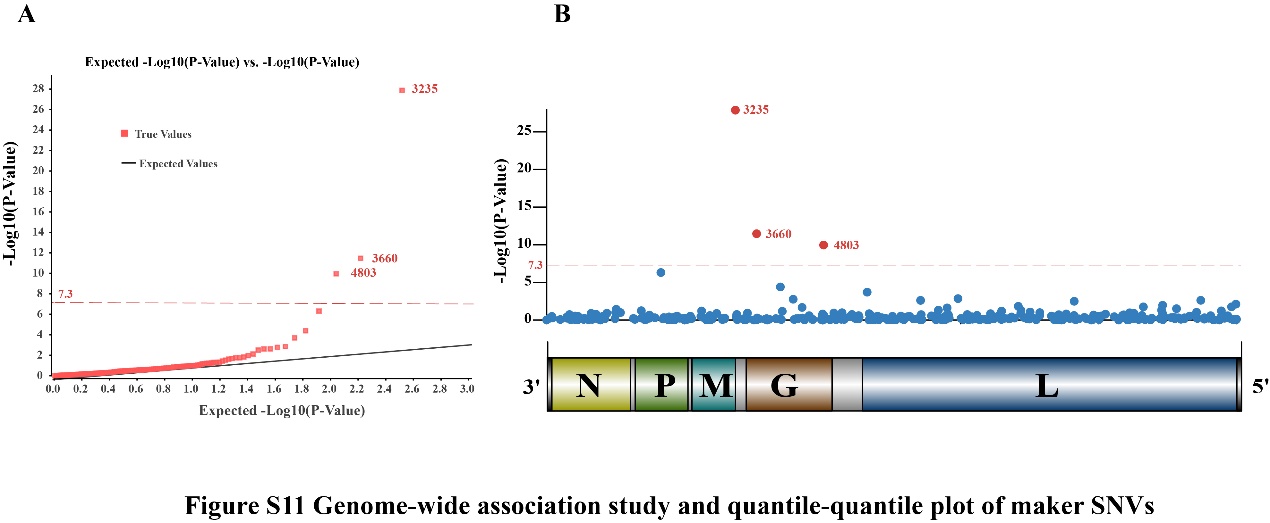


**Figure S11 Genome-wide association study and quantile-quantile plot of maker SNVs.**

There SNVs, 3225, 3660(G115), 4803(G496) were identified with –lg (p values) over 7.3, implying the mutations might be associated with the traits.


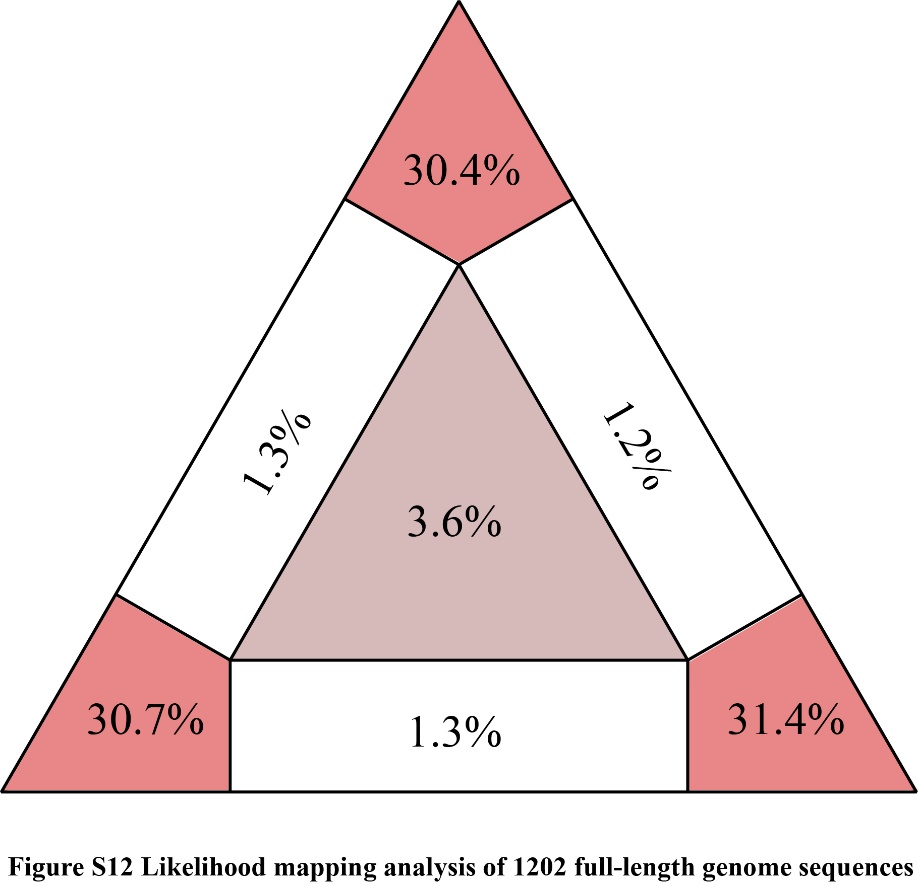


**Fig. S12 Likelihood mapping analysis of 1202 full-length genome sequences.**

Quartets falling into the three corners are informative. Those in three rectangles are partly informative and those in the center are uninformative.
